# Haemodynamic Wall Shear Stress, Endothelial Permeability and Atherosclerosis—A Triad of Controversy

**DOI:** 10.3389/fbioe.2022.836680

**Published:** 2022-03-07

**Authors:** Peter D. Weinberg

**Affiliations:** Department of Bioengineering, Imperial College London, London, United Kingdom

**Keywords:** Hemodynamics, endothelial cell, OSI, transWSS, vesicles, follistatin-like 1, FSTL1, inflammation

## Abstract

A striking feature of atherosclerosis is its patchy distribution within the vascular system; certain arteries and certain locations within each artery are preferentially affected. Identifying the local risk factors underlying this phenomenon may lead to new therapeutic strategies. The large variation in lesion prevalence in areas of curvature and branching has motivated a search for haemodynamic triggers, particular those related to wall shear stress (WSS). The fact that lesions are rich in blood-derived lipids has motivated studies of local endothelial permeability. However, the location of lesions, the underlying haemodynamic triggers, the role of permeability, the routes by which lipids cross the endothelium, and the mechanisms by which WSS affects permeability have all been areas of controversy. This review presents evidence for and against the current consensus that lesions are triggered by low and/or oscillatory WSS and that this type of shear profile leads to elevated entry of low density lipoprotein (LDL) into the wall via widened intercellular junctions; it also evaluates more recent evidence that lesion location changes with age, that multidirectional shear stress plays a key role, that LDL dominantly crosses the endothelium by transcytosis, and that the link between flow and permeability results from hitherto unrecognised shear-sensitive mediators.

## Introduction

A striking feature of atherosclerosis is its non-uniform distribution within the arterial system. Some arteries, and some regions within individual arteries, remain free of disease even when others have developed it to a life-threatening extent (Mitchell and Schwartz, 1965). This feature implies the existence of powerful local risk factors. The identification of global risk factors such as hypertension and hyperlipidaemia has led to the development of therapies that significantly reduce the prevalence of the disease and its sequelae. The identification of local risk factors has been mired in controversy and currently no therapies are based on them.

An early hypothesis (Anitschkow, 1933) was the causal chain:

mechanical factors→enhanced uptake of circulating lipids into the wall→inflammation→disease.

A more modern addition to this insudation theory or lipid hypothesis is that the lipid needs to be modified in some way—perhaps by oxidation, aggregation or both (Quinn et al., 1987; Williams and Tabas, 1995; Javed et al., 1999; Wen and Leake, 2007; Wen et al., 2019)—before an inflammatory response occurs. These ideas underlie the use of statins to control the development of the disease, and an extensive search for therapeutic effects of dietary antioxidants.

Despite apparent acceptance of the insudation theory, there has been debate about where lesions occur, the stresses responsible, the direction of lipid transport that is critical, the mediators linking mechanical stresses to transport, and the nature of the transport pathway. In order to derive new therapies that confer on disease-prone arteries the properties of disease-resistant ones, resolution of these controversies is required. Misidentification of, say, the mechanical triggers or the relevant transport route could lead to the design of ineffective interventions targeting inappropriate pathways. This review provides an overview of the debates and introduces recent developments in the field that suggest novel targets. It is structured and summarised as follows:

### Historical Background

- Early studies showed that cholesterol-fed rabbits develop lesions downstream of aortic branch points; the pattern was explained by high haemodynamic wall shear stress (WSS) elevating endothelial permeability to circulating lipoproteins. In people, lesions were later found to occur upstream of branches; the human pattern was attributed to low and oscillatory WSS and could not be explained by elevated permeability.

### Contradictions Can Be Resolved by Taking Age Into Account

- Patterns of lesions in rabbits and people switch location with age, and in the same way. The previous contradiction is attributed to immature rabbits being inappropriately compared with mature people. Age-related patterns of insudation can explain both patterns.

### The Emergence of Other Disease Patterns

- Lesions occur not only upstream and downstream of branches: intermediate patterns are seen and there may be a series or continuum of changes with age.

### Underlying Mechanisms

- Many other types of mechanical stresses have been implicated in atherogenesis. Is it the relation between mechanical forces and atherogenesis that changes with age, or is it the mechanical forces themselves that change? Flow-dependent NO release and its effect on permeability appear to change with age, supporting the former, but the pattern of endothelial morphology also changes, supporting the latter.

### Computational Studies of Flow

- Numerical simulations have been used to further investigate if flow patterns change with age. They provide little evidence that time average or oscillatory WSS change substantially.

### Potential Importance of Multidirectional Flow

- A systematic review showed that the strength of evidence for the low WSS theory is not as strong as widely supposed. The pattern of an alternative WSS metric, the transverse WSS, changes with age and correlates much better with lesion location and elevated permeability.

### Further Investigation of Mechanisms Relating Flow to Permeability

- *In vivo* studies employing modern techniques suggest that “hotspots” of high endothelial permeability account for only a small fraction of uptake, at least for albumin-sized molecules, and *in silico s*tudies that take into account the presence of the glycocalyx suggest that concentration polarisation at the endothelial surface also has a limited effect. Recent *in vitro* studies suggest transendothelial transport of the largest macromolecules occurs by transcytosis and that it is increased by multidirectional flow.

### Proposal of a Secreted Mediator

- Endothelial cells exposed to uniaxial flow *in vitro* release a soluble mediator that suppresses transcytosis of LDL. Cells exposed to multidirectional flow do not. The mediator also has anti-inflammatory effects.

The review finishes with three sections that 1) discuss limitations to the above synthesis, 2) present perspectives and suggest future work, and 3) draw conclusions.

## Historical Background

Statements about mechanical stresses and atherosclerosis appear in the 19th century (Rindfleisch, 1872) but the experimental study of localising factors began in the early 20th century with the work of Anitschkow (alt.—Anichkov, Russian—Ани́чков) and his colleagues. A retrospective review of that work (Anitschkow, 1933) remains of interest today for its scientific content, as well as for its historical significance.

The first step by the St Petersburg group was the development of the cholesterol-fed rabbit model. The Russian school at that time held that atherosclerosis was a natural consequence of aging, and also that aging was accelerated by eating meat. Rabbits fed meat did indeed develop an atherosclerosis-like disease (Steinberg, 2013). Anitschow showed, however, that it was the cholesterol in the meat that was responsible: rabbits fed diets supplemented only with cholesterol also developed lesions.

**FIGURE 1:**
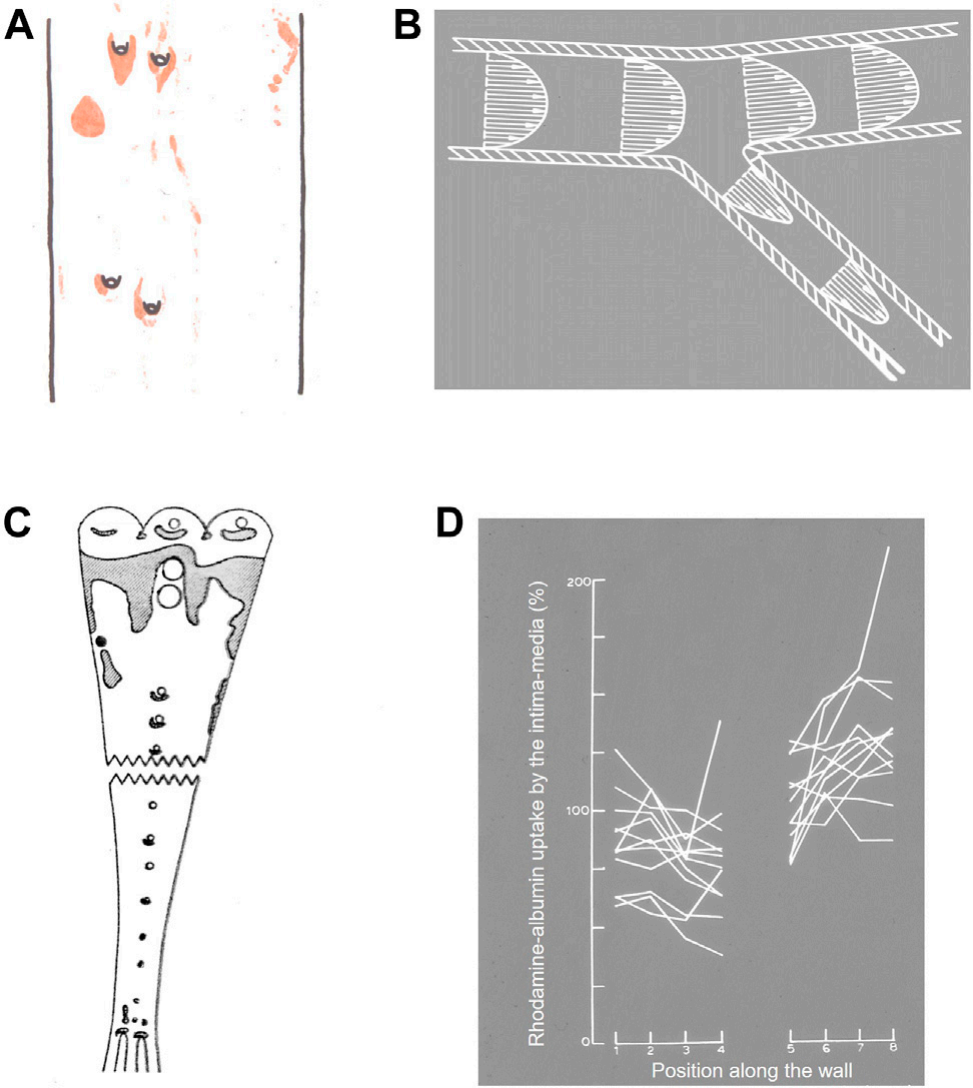
**(A)** Watercolour from Anitschkow (1933) of lipid staining (red) near four intercostal branch ostia in a cholesterol-fed rabbit. The thoracic aorta is viewed *en face* and mean aortic flow is from top to bottom. **(B)** Conceptualisation of velocity vectors and velocity profiles in a 2-D model of a branching artery, after Fry (1969). **(C)** Uptake of Evans’ Blue Dye (shaded areas) in the aorta of a pig (Somer and Schwartz, 1971). Note that pigs have unpaired intercostal ostia, unlike the paired ostia in human and rabbit aortas. The aorta is viewed *en face* and mean aortic flow is from top to bottom. **(D)** Uptake of rhodamine-labelled albumin into the aortic wall of rabbits up to approximately one branch diameter upstream (positions 1→4) and downstream (positions 5→8) of 12 intercostal ostia (positions 4→5), 3 h after the tracer was administered (Weinberg, 1988).

Anitschkow (1933) drew particular attention to the remarkable pattern of lipid staining seen around the ostia of side branches of the aorta. Lesions occurred in an arrowhead pattern surrounding the downstream half of the origins of the intercostal arteries (Figure 1A). These branch points have subsequently received much study and are the main focus of the present review. (Supplementary Material Figure S1 shows the location of each arterial region discussed in this review; the large-scale structure of the normal and diseased artery wall (Pang et al., 2021b) is shown in Supplementary Material Figure S2.)

It had been known since the 1850s, when microscopes were applied to pathology, that the lipid deposition in atherosclerosis occurs within the wall rather than on its surface. Anitschkow reasoned that the dietary cholesterol entered the bloodstream and that the patchy nature of the disease resulted from a patchy entry of circulating “lipoids” into the wall, as part of a process similar to the one in which lymph is formed by filtration of plasma across capillary walls. This hypothesis was supported by experiments that introduced the intravital dye Trypan Blue into the circulation of frogs. Trypan Blue, like its isomer Evans’ Blue Dye (EBD), binds to circulating proteins, particularly albumin, and is carried into the wall when they enter it, leading to a blue colouration in areas where entry is particularly rapid. Petroff (1922) observed blue patches on the flow divider of branch points in large mesenteric vessels. Anitschkow (1933) suggested that mechanical forces led to this spatial variation in wall transport properties, but did not speculate further.

It was Fry (1969) who extended this view by proposing that the mechanical trigger is a pathologically elevated level of haemodynamic wall shear stress, the force per unit area acting parallel to the endothelium as a result of the flow of blood. His fluid mechanical argument is shown in Figure 1B. For simplicity, blood was assumed to be a continuous fluid with Newtonian rheology, flow to be steady, walls to be rigid and the geometry to be two-dimensional. Upstream of the side branch, the velocity profile is at least partially developed—that is, layers at considerable distances from the wall are slowed by viscous drag—and WSS, which is proportional to the near-wall velocity gradient, is therefore relatively low. It is the slower moving blood near the wall that enters the branch, while more rapidly moving blood, previously near the centre of the vessel, impinges on the flow divider of the branch. This fast-moving blood itself gets slowed by viscous interactions as it moves downstream but, in the region before this occurs, the velocity gradient and hence WSS are elevated. WSS would, by the same argument, be relatively high on the flow divider of a symmetrical bifurcation and lower on the outer (“lateral”) walls, especially if separation were to occur.

Fry (1969) acutely exposed the luminal surface of arteries to various fluid mechanical stresses and found that WSS magnitudes above 400 dyn/cm^2^ injured and, at a sufficiently high level, even removed the endothelial cells, leading to elevated influx of EBD-labelled albumin and, it was assumed, the larger lipoproteins.

Subsequent publications from Fry and colleagues recognised greater complexity: that different endothelial responses might occur over different time courses, that the structure of the underlying wall might influence the response, and that subtle features of branch geometry might have profound influences on the distribution of WSS (e.g. Fry, 1973). Nevertheless, the original hypothesis was intuitively satisfying and became the consensus view. It was supported by much subsequent data. Quantitative mapping of lesions in rabbits fed a high cholesterol diet for short periods or a lower cholesterol diet for longer periods, or in rabbits that were hypercholesterolaemic as a result of genetic mutations, all confirmed the Anitschkow pattern of lipid deposition in the aortic wall around ostia of side branches (Cornhill and Roach, 1976; Roach et al., 1978; Forster et al., 1996). Studies with intravital dyes confirmed that elevated uptake occurrs in the equivalent regions in the pig (Somer and Schwartz, 1971; Figure 1C). Such studies can be criticised: EBD binds to elastin (Adams, 1981) and collagen (Heinle and Lindner, 1984) once in the wall and its concentration may therefore be affected by variation in wall structure as well as in transport, whilst a fraction of the dye circulates in its free form (Lindner and Heinle, 1982) and may enter the wall by routes not available to macromolecules. However, quantitative measurements of transport using radiolabelled low-density lipoprotein (LDL) (Schwenke and Carew, 1988) or albumin covalently labelled with a fluorescent dye (Weinberg, 1988) showed that net uptake of these circulating macromolecules was likewise elevated downstream of branches in the rabbit aorta (Figure 1D), even though elevated EBD accumulation is hard to demonstrate in this species (Friedman and Byers, 1963).

**FIGURE 2:**
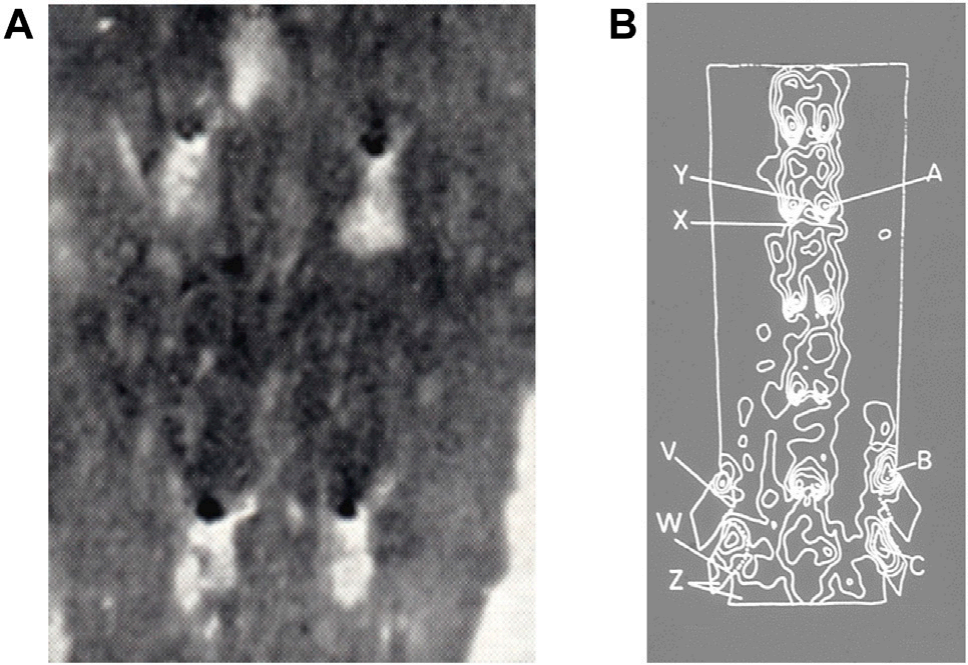
**(A)** Lipid staining (black) near four intercostal branch ostia in an adult human aorta, viewed *en face* with mean aortic flow from top to bottom (Mitchell and Schwartz, 1965). **(B)** Contour map of lesion prevalence in the human aorta, opened ventrally and viewed *en face* with mean aortic flow from top to bottom. Flow dividers on the dorsal wall are indicated by bright “horseshoe” shapes. Prevalence is high at point A and low at point X (Svindland and Walløe, 1985).

Despite this wealth of evidence, issues arose in applying Fry’s high-shear hypothesis to human disease. Mitchell and Schwartz (1965) had already noted a different distribution of lipid staining in *post mortem* human specimens: Sudanophilia occurred in a dorsal streak in the descending thoracic aorta but there was striking sparing around and immediately distal to the ostia of the intercostal arteries (Figure 2A). Caro et al. (1971) subsequently quantified the distribution in the initial segment of the large branches of the human abdominal aorta, finding that fatty streaks were less prevalent in the quadrant containing the flow divider than in other quadrants. These distributions of human disease were confirmed and extended by Svindland and Walloe (1985; Figure 2B) and by Cornhill et al. (1990). Disease was again found in the inflow tract of large abdominal branches but it was also shown in the aortic wall upstream of the branches. In the thoracic segment, lipid staining was found to be maximal upstream of intercostal ostia and low distal to them.

From Figure 1B, it is apparent that WSS should be elevated on both the aortic and the branch side of the flow divider. Caro et al. (1971) therefore concluded from their study of lesions in the initial segment of branches that high WSS protects against the development of human disease, which instead occurs in areas of low WSS. Note that the disagreement with the high WSS hypothesis of Fry concerns the location of lesions, not the nature of local blood flow.

The low shear hypothesis of Caro et al. has been modified somewhat. Ku et al. (1985) compared the distribution of intimal thickening in the human carotid bifurcation with laser Doppler measurements of flow in anatomically realistic models of the same specimens. This work showed that intimal thickening was highest in regions where not only was time average WSS low but values of a metric called the Oscillatory Shear Index (OSI) were high. Indeed, the correlation with the OSI was stronger than with mean WSS.

Despite its name, the OSI captures any instantaneous WSS vector that is not aligned with the mean WSS vector and not just those that are at 180° degrees to it, as in oscillatory flow. As later defined by He and Ku (1996):

$$\mathrm{OSI}=\frac{1}{2}\left(1-\frac{\left|\int_{0}^{T}{\vec{\tau }}_{w}dt\right|}{\int_{0}^{T}\left|{\vec{\tau }}_{w}\right|dt}\right)=\frac{1}{2}\left(1-\frac{\left|{\vec{\tau }}_{mean}\right|}{\mathrm{TAWSS}}\right)\;\mathrm{where}\;{\vec{\tau }}_{mean}=\frac{1}{T}\int_{0}^{T}{\vec{\tau }}_{w}dt$$

and ${\vec{\tau }}_{w}$ represents the instantaneous WSS vector, t the time and T the duration of the cardiac cycle.

The OSI tends to be high where time average WSS is low and *vice versa*. The two hypotheses have thus been conflated to some extent.

Today, the low/oscillatory WSS hypothesis is the consensus view; the paper of Caro et al. (1971), now 50 years old, is a citation classic. However, the hypothesis took many years to become widely accepted. This probably reflects the contradiction with the results of experimental studies in animals, particularly rabbits, where the location of lesions is not only different but correlates with the pattern of elevated uptake of plasma macromolecules by the wall. Caro et al. (1971) assumed that the same pattern of uptake occurred in people. To account for the discrepancy in lesion location, they had to assume different mechanisms: that plasma cholesterol concentrations were higher than wall concentrations in the rabbit, and disease therefore occurred where transport between the two compartments was fastest, whereas concentrations of cholesterol or a modified, atherogenic version of it were higher in the wall than in plasma in people, meaning that disease would occur where transport between the two compartments was slowest. This concept predicts mirror-image patterns of disease in the two species.

A corollary of the proposal, developed in a mathematical appendix to the paper, is that the level of disease in people must be independent of plasma cholesterol concentrations. Although that might have been tenable in 1971, many subsequent studies have demonstrated a link and the more recent success of cholesterol-lowering statins has made the connection practically incontrovertible. Thus there is a fundamental flaw at the centre of our understanding of atherogenesis: the accepted haemodynamic explanation for the localisation of the disease is incompatible with the most widely used therapy and the insudation theory on which it is based.

Rabbit models of atherosclerosis have been superseded to a large extent by genetically modified mouse models. Furthermore, although Anitschkow’s introduction of the cholesterol-fed rabbit is eulogised by many (e.g. Steinberg, 2004), it has been anathematised by others (e.g. Stehbens, 1999). Patterns of disease and transport obtained in the rabbit are not widely discussed at present and the central inconsistency emphasised above is rarely mentioned. However, rabbits are phylogenetically the closest species to human beings outside of the primate order. Also, many haemodynamic stresses and non-dimensional groups obey allometric scaling laws and the discrepancy in their value between mice and people is therefore much greater than the discrepancy between rabbits and people. Aortic WSS, for example, depends on body mass raised to the inverse 3/8ths power (reviewed in Weinberg and Ethier, 2007) and is thus 20-fold higher in mice than in people. Ignoring findings obtained in rabbits may be unwise.

Caro and colleagues themselves made an attempt to resolve the discrepancy. To understand their reasoning, it is first necessary to introduce some fundamentals of wall mass transport. There is a pressure gradient from the arterial lumen to the adventitia, driving a constant convective flow across the wall. Dissolved macromolecules will be advected in the same direction, but may be filtered at various interfaces though which water can move more readily than the solute. At such points, solute concentration will rise, like a filter cake, until it drives sufficient diffusion in the reverse direction to match the forward advective flux. This phenomenon is known as concentration polarisation. There have been many studies of its putative occurrence at the endothelial surface, but additional concentration polarisation may occur within the wall. For example, LDL concentrations in the arterial intima appear to be higher than those in plasma and this has been attributed to filtering by the internal elastic lamina (Smith and Staples, 1980).

Caro and Lever (1983) suggested that intimal trapping might also arise if the arterial media restricted transport of large solutes more than water. A corollary is that the degree of trapping might depend on the tone of medial smooth muscle cells, and hence could vary according to the mix of vasoactive agents present. Medial tone, by affecting porosity, would also influence the space available for solutes and hence the amount of solute present in each volume of wall tissue. Thus it is conceivable that the intimal concentration of an atherogenic macromolecule could be high in areas where the level averaged over the thickness of the wall is low; both could be caused by low medial porosity.

A phenomenon of this type might explain an apparent inverse relation between local wall uptake of plasma macromolecules and the predilection for lesions, avoiding the need to invoke transport from the wall into the lumen, with its attendant problems. However, it cannot be a complete solution to the incompatibilities noted above. First, the binding of EBD to elastin means that it should be an excellent indicator of the rate of entry of proteins into the wall, and that it should be little affected by the space available for macromolecules in the media. Second, the different sites of lesions in rabbit and human arteries remain unexplained. Like the first mechanism of Caro et al. (1971), this second attempt has not entered the mainstream. We are thus left with a paradigm in which low shear stress leads to dysfunctional, leaky endothelium and hence to disease, even though the predicted patterns of shear stress and measured patterns of permeability in the rabbit contradict it.

## Contradictions Can Be Resolved by Taking Age Into Account

A potential resolution of the apparent contradictions described above arises from the observation that anatomical patterns of transport and disease change with age. The fundamental suggestion is that immature rabbits have been inappropriately compared with mature people. Rabbits tend to be used when young because it is costly to house them for long periods. *Post mortem* human arteries generally derive from older, or at least mature, populations. When age is taken into account, there is a strong spatial correlation between sites of rabbit permeability, rabbit lesions and human lesions.

**FIGURE 3:**
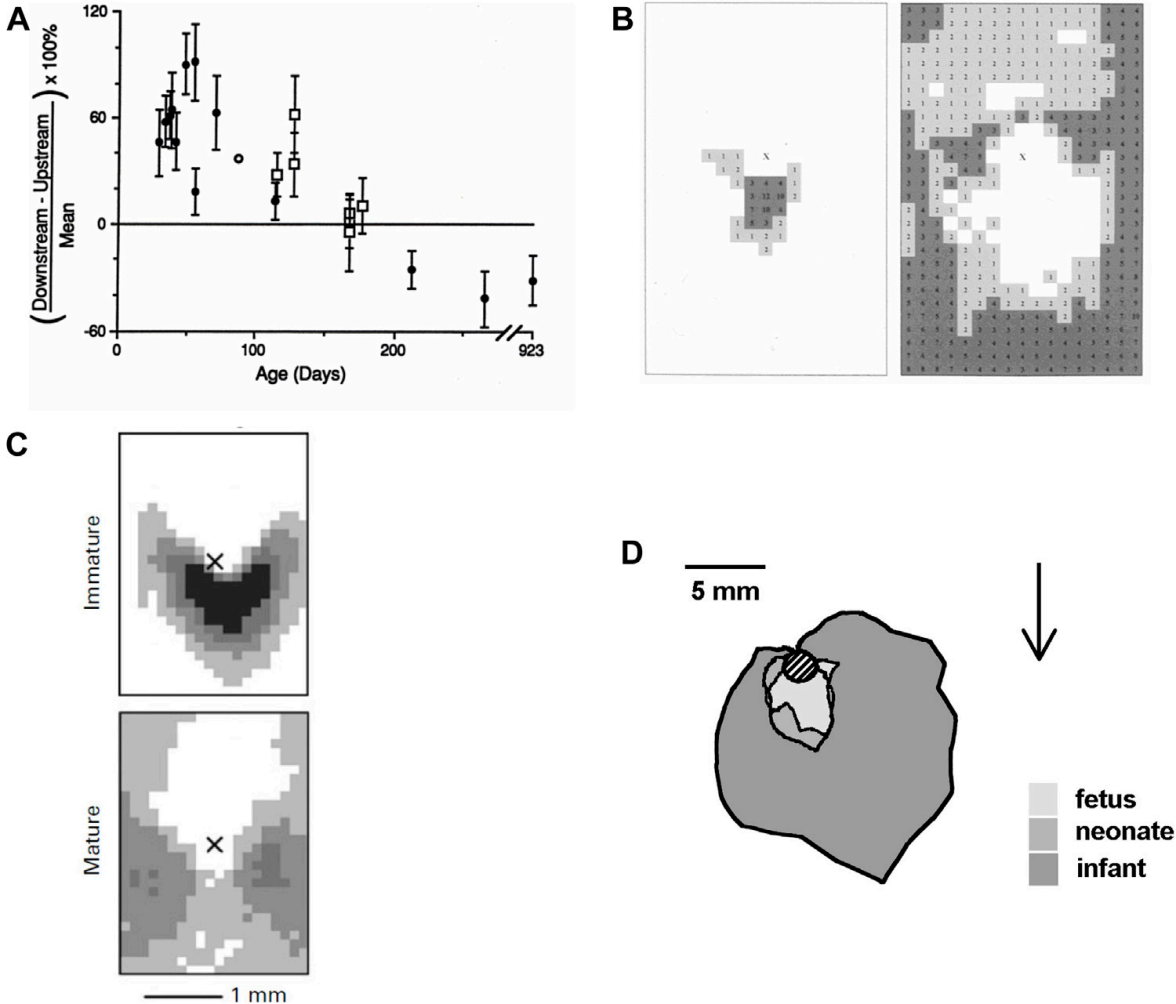
**(A)** Difference between uptake of albumin downstream and upstream of intercostal branch ostia, expressed as a percentage of the mean uptake in both regions. Uptake is greater downstream in young rabbits and greater upstream in older rabbits (Sebkhi and Weinberg, 1994a). **(B)** Maps showing the prevalence of spontaneous lipid deposition around intercostal branch ostia in weanling (left) and aged (right) rabbits fed a normal diet. The centre of the ostium is marked with an “X,” mean aortic flow is from top to bottom and increased lesion prevalence is indicated by numbers (% of branches affected at each site) and by darker shading (Barnes and Weinberg, 1998). **(C)** Similar maps of lesion prevalence in immature and mature rabbits fed a cholesterol-enhanced diet (Cremers et al., 2011). **(D)** Average extent of lesions from the lip of intercostal ostia (shaded circle) in human fetuses, neonates and infants up to the age of 1 year. Arrow shows mean flow direction (after Sinzinger et al., 1980).

The first study (Sebkhi and Weinberg, 1994a) in the development of this concept, conducted nearly 30 years ago, showed that net uptake of circulating albumin by the aortic wall was greater downstream than upstream of intercostal branch ostia in immature rabbits, but greater upstream than downstream in mature rabbits. The transition occurred at around 6 months of age (Figure 3A). Mean uptake did not change, so uptake must have been decreasing downstream and increasing upstream with age. (There was a sharp drop in mean uptake shortly after weaning.)

This study assessed uptake 3 h after tracer administration. At this time, levels of labelled albumin in the wall are approaching a steady state, where they should resemble those of the native protein. Steady-state levels of an inert particle reflect three properties: the ease of entry into the wall (here termed permeability, regardless of the mechanism of entry), the ease of egress from the wall, and the space available for the particle within the wall.

A subsequent study (Sebkhi and Weinberg, 1996) examined uptake 10 min after tracer administration. After such a short period, concentrations of tracer in the space available to it are much lower than concentrations in plasma, and will be dominantly determined by ease of entry. The same age-related patterns were found, supporting the view that permeability is the limiting factor. Murphy and Lever (2002) similarly found that short-term uptake of labelled albumin was greater downstream than upstream of the renal artery in immature rabbits and showed the reverse pattern in mature ones. (However, they were unable to demonstrate a parallel pattern of quasi-steady uptake in mature animals.)

Spontaneous lipid deposition in rabbit arteries is rare, but it can be detected in weanling and old animals. A study (Barnes and Weinberg, 1998) mapping lipid deposition in rabbits of these ages found an arrowhead distribution downstream of intercostal branch ostia in the weanlings and sparing of this area in old animals, where lipid staining instead occurred further downstream and at the sides of the branch (Figure 3B). A trend from a downstream arrowhead to a pattern with more lateral and upstream lipid deposition was also seen at the origin of the coeliac artery. (The fact that disease developed at all in the mature animals is a challenge to the insudation theory because their average plasma cholesterol concentration was only 18 mg/dl).

The patterns in cholesterol-fed rabbits proved harder to elucidate. A small study (Sebkhi and Weinberg, 1994b) of quasi-steady albumin uptake showed that feeding mature rabbits 0.2% cholesterol for 1 week reversed the usual pattern of transport, so that uptake became greater downstream than upstream of intercostal branch ostia, as in immature animals. However, after a further week of feeding, the pattern reverted to normal. This made it uncertain whether to expect the downstream pattern of lesions if mature rabbits were fed cholesterol, or one where the area downstream of branches was spared.

In fact, both patterns—and intermediates between them—were observed. In a first trial (Barnes and Weinberg, 1999), lesions occurred in the classical Anitschkow arrowhead around the downstream margin of intercostal branch ostia in the aortas of both immature and mature animals. In a second trial (Barnes and Weinberg, 1999), mature animals had lesions lateral to the branch ostia with areas upstream and downstream of the branch being spared. The discrepancy was attributed to differences in the diet, especially since adding vitamin E to the diet in the second trial, with the aim of preserving the nitric oxide pathway, increased disease upstream of the branch.

This idea was explored in a third trial (Barnes and Weinberg, 2001) by additionally giving the cholesterol-fed rabbits either vitamin E, or L-arginine or L-*N* ^G^-nitro-arginine methyl ester (L-NAME) which are, respectively, the precursor and an inhibitor of NO production. All the diets gave a pattern that was intermediate between the two previously observed: lesions occurred downstream of the branch except along a line extending axially from the branch centreline, as if the downstream arrowhead were splitting into two lobes. A fourth trial (Barnes and Weinberg, 2001) examined the dietary fundamentals by using either of the two base diets and either of the two cholesterol regimens (0.2% for 15 weeks or 1% for 8 weeks) previously employed. Both the downstream and the lateral pattern were observed, but neither base diet and neither cholesterol regimen consistently gave one or the other.

The fourth trial was the first in which lesion patterns differed substantially between and within groups and was also the first in which it had not been possible to obtain all the rabbits from a single supplier. This motivated a retrospective analysis (Barnes and Weinberg, 2001) of mature animals from all four trials; it showed that the discrepancies could be explained completely by which strain of rabbit had been used. Although all rabbits were New Zealand Whites and >11 months old, one strain consistently showed the downstream triangle, a second consistently showed the lateral pattern, and two gave the intermediate pattern. A subsequent trial (Cremers et al., 2011) in which immature and mature rabbits of the second strain were given the same diet for the same length of time unequivocally gave the arrowhead pattern at intercostal branch ostia of the immature animals and a more lateral pattern in the mature ones (Figure 3C).

To confirm that this unexpected effect of strain applies to the pattern of transport as well as lesions, short-term uptake of albumin was examined in two strains of rabbit (Staughton and Weinberg, 2004a). Both showed a gradual change with age from uptake being greater downstream of the branch to it being greater upstream of the branch, as before. However, the age at which the pattern switched—i.e. at which uptake was equal in both regions—was approximately 650 days in one strain and 1,300 days in the second. These values were significantly different, and both were markedly different to the approximately 180 days observed in the initial studies described above, which used a third strain of rabbit. Hence the switch does not necessarily occur at the age of sexual maturation, as originally supposed.

An interesting additional finding of this study was the existence of a circadian effect: the pattern of uptake was consistently biased towards the upstream pattern in the morning, compared to the afternoon. This suggests that the pattern depends in part on a physiological property that can vary over a matter of hours, in addition to any influences of more time-invariant properties such as arterial geometry.

Do parallel age-related changes occur in people? There is one quantitative study mapping the distribution of lipid deposits in *post mortem* aortas of human fetuses, neonates, and infants below the age of 1 year (Sinzinger et al., 1980). Sudanophilia was found exclusively around the downstream margins of branch ostia in the youngest aortas, and all around the branches but with highest prevalence downstream, in the older ones (Figure 3D). Although the authors explicitly noted that this pattern is at variance with the one obtained in adult vessels and is similar to data for the cholesterol-fed rabbit, the work has received only 5% of the citations of the paper of Caro et al. (1971), presumably because it does not fit the current consensus.

The important result of Sinzinger et al. is consistent with qualitative data from much earlier studies. Zinserling (1925) noted the presence of small, distinct spots of lipid staining, one below each intercostal ostium, and lesions in the form of a Y-shaped fork, with two stripes that join under the ostium, in children. Thus there appears to be a switch with age in the distribution of lipid deposits in the human aorta that parallels the one observed in the rabbit. It is interesting to speculate that, as with rabbits, different populations may undergo the swich at different ages.

### Conclusion

The data described in this section eliminate the need to regard the cholesterol-fed rabbit model as aberrant in some way. On the contrary, both spontaneous and diet-induced lesions in the rabbit seem to replicate closely an age-related switch, largely ignored, that occurs in human vessels. Furthermore, the rabbit provides a mechanism: wall uptake of circulating macromolecules shows the same age-related switch. The permeability of the wall appears to be the critical property.

There is no need to postulate that transport out of the wall is critical, with the attendant problem in accounting for the dependence of disease on plasma cholesterol concentrations: regions downstream of branches can be diseased or spared in animals consuming diets supplemented with the same high level of cholesterol for the same duration (Cremers et al., 2011). Equally, there is no need to postulate that disease is triggered by variation in intimal macromolecule accumulation that has an undetected anatomical distribution opposite to that seen in the wall as a whole. Indeed, when uptake of albumin measured over the entire intima-media is higher downstream of branches than upstream, then the same is true for uptake in the innermost layers of the wall; there is no indication of a reversed pattern in the innermost layers that could account for an upstream distribution of lesions (Sebkhi and Weinberg, 1996).

Provided that age is taken into account, the simple insudation theory can account for the pattern of human lesions, as well as rabbit lesions. Indeed, the fact that patterns of transport and lesions remain correlated despite both changing with age greatly strengthens the insudation theory; there are many properties that vary around arterial branches, but presumably far fewer that show the necessary switch with age. The changes with age add complexity but, paradoxically, make it easier to identify localising factors.

## The Emergence of Other Disease Patterns

In the preceding sections, lesion prevalence and permeability have been discussed largely in a binary fashion: are they greater upstream or downstream of branch ostia? However, the patterns are more complex and more numerous than this, which has implications for the mechanisms involved.

**FIGURE 4:**
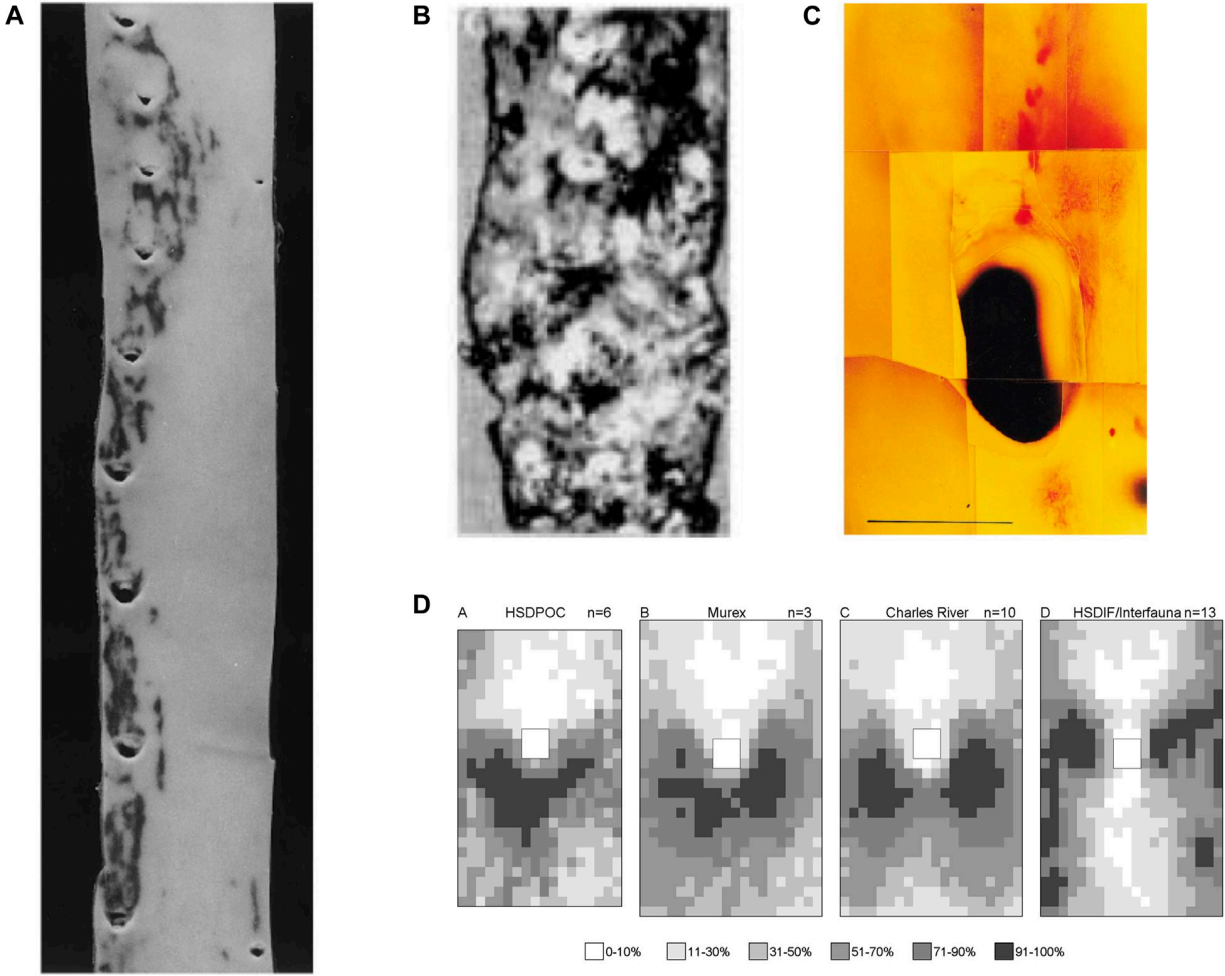
**(A)** Human thoracic aorta, viewed as in Figure 2A, showing two patterns of lipid staining at intercostal branch ostia (Sloop et al., 1998). **(B)** Lesions in an aged human aorta, viewed as in Figure 2A. Fibrous caps of raised lesions appear white and can be seen completely surrounding the dark holes of branch ostia (Mitchell and Schwartz, 1965). **(C)** Spontaneous lipid deposition (red) extending upstream of the coeliac branch ostium in the abdominal aorta of an aged rabbit fed a normal diet (Barnes and Weinberg, 1998). **(D)** Maps of lesion prevalence, displayed as in Figure 3C, in mature rabbits of four different strains. The maps are arranged in an order that gives the impression of a trend from the downstream arrowhead to the lateral pattern of disease (Barnes and Weinberg, 2001).

Sloop et al. (1998) demonstrated the existence of two patterns of fatty streaks in adult human aortas. In one pattern, lesions occurred lateral to intercostal branch ostia, and in longitudinal streaks joining those areas. In a second pattern, lesions extended proximally from the inflow tract of intercostal ostia. Occasionally, both patterns were seen in a single aorta (Figure 4A). The average age of subjects with the first pattern was significantly lower than those with the second pattern, although the difference was remarkably small (20.5 ± 3.5 vs 25.8 ± 1.3 years, *p* = 0.004). Subjects with equal prevalence of both patterns had an intermediate age (23.5 ± 2 years). The only other variable associated with the switch was the serum isothiocyanate level, indicative of smoking.

Mitchell and Schwartz (1965) found that raised lesions in human aortas completely surround branch ostia, giving the appearance of a volcano (Figure 4B). The discrepancy with the pattern of lipid deposition was used to argue that fatty streaks are not precursors of raised lesions. Sloop et al. (2002) have made the same argument based on the development of raised fibroproliferative lesions in synthetic dialysis access grafts, without prior fatty streaking. This view has not been widely reported or accepted.

Thus in people, there are downstream, lateral, and upstream fatty streaks, and raised lesions that surround ostia. There appears to be a progression in that order with increasing age. The first two have been demonstrated unequivocally in immature and mature rabbits, while the third may occur spontaneously at the coeliac branch ostium in aged animals (Figure 4C). The fourth has not been quantified in rabbits but example images of raised lesions in hares fed cholesterol-supplemented and normal diets, alternating every week for up to 18 months (Imai and Lee, 1983), do appear to show disease at several points around aortic branches.

Is there a continuous change in lesion pattern or a series of discrete switches from one to another? Although upstream streaks are visible at distal branches of the human aorta shown in Figure 4A, and lateral streaks at the proximal branches, branches at the centre of the image appear to show transitional forms. Similarly, the patterns of lesions observed in mature, cholesterol-fed rabbits of different strains, described above, can be arranged in a sequence (Figure 4D) that—although not corresponding to chronological age—supports the view that there is a smooth transition from the downstream to the lateral pattern rather than an abrupt switch.

Extending the argument made above, the multiplicity of lesion patterns should make the identity of a mechanism, if there is a single one, even more secure. Can permeability of the arterial wall account for all the patterns? In the studies discussed above, uptake of fluorescently-labelled albumin by the wall upstream and downstream of branches was measured in histological sections cut along the aortic axis, through the centreline of the branch. Such measurements cannot rigorously be compared to the various patterns of lesions. Indeed, the centreline through the branch defines a plane of symmetry that may behave quite differently from planes displaced even a short distance to either side.

A study (Ewins et al., 2002) that laboriously cut serial longitudinal sections across the width of the branch ostium indicated that the “downstream greater than upstream” pattern of permeability in immature rabbits was in fact a downstream arrowhead, whereas the “upstream greater than downstream” pattern in mature rabbits actually had highest uptake at the lateral margins of the branch and in streaks extending upstream and downstream from this location.

**FIGURE 5:**
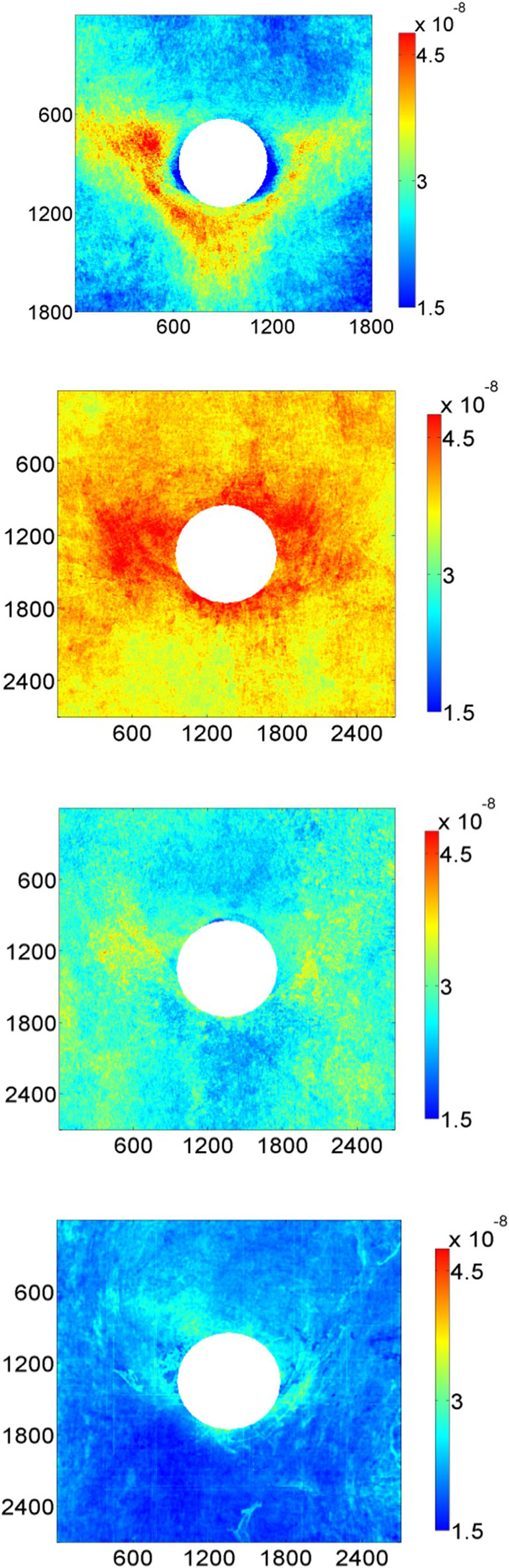
Maps of 10-minute albumin uptake by the aortic wall of rabbits around intercostal branch ostia. The four panels represent, from top to bottom, rabbits aged 9 weeks, 6 months, 16–22 months and >5 years (Bailey et al., 2015). Uptake is expressed as mass transfer coefficients (cm/s), mean aortic flow is from top to bottom and dimensions are in μm.

The subsequent development of rapid detection techniques based on confocal microscopy (Clarke et al., 2012) allowed 3-dimensional mapping of tracer uptake around the branch at a range of ages (Bailey et al., 2015). Maps showed a transition from the downstream triangle via an intermediate form to a lateral pattern, and then to an upstream pattern in animals >5 years old (Figure 5). (A reduced analysis of these four patterns showed a continuous increase in the ratio of transport directly upstream of the branch to that directly downstream.) The agreement with the distribution of fatty streaks in rabbits and people seems excellent. The mechanism underlying the development of raised lesions requires further investigation; note that such disease may affect its own progression by disturbing blood flow. An interesting feature of the data is that branches on the left and right side of the aorta showed slightly different patterns of uptake, consistent with the patterns being determined not just by the branch itself but also by larger-scale flow features.

## Underlying Mechanisms

Anitschkow (1933) attributed the non-uniform pattern of transport and lesions to variation in mechanical forces. Three putative triggers have already been mentioned. They all concern WSS, although they differ as to whether it is high (Fry, 1969), low (Caro et al., 1971) or oscillatory shear (Ku et al., 1985) that is critical. Other putative triggers involving flow include turbulence (Wesolowski et al., 1965), fluctuating WSS (Fry, 1973), pulse WSS (Friedman et al., 1981), temporal WSS gradient (Bao et al., 1999), WSS harmonic content (Himburg and Friedman, 2006), spatial WSS gradient (Lei et al., 1995), WSS angle gradient (Kleinstreuer et al., 2001), the WSS angle deviation (Hyun et al., 2000), multidirectional WSS at stagnation points (McMillan, 1985a; McMillan, 1985b), multidirectional WSS generally (Peiffer et al., 2013a; Mohamied et al., 2015), particle residence time (Moore Jr et al., 1992) and relative residence time (Himburg et al., 2004). The plethora of hypotheses may explain why vascular biologist frequently use the vague and hence undesirable term “disturbed flow” to indicate atherogenic stresses.

Spatial variation in pressure, the stress normal to the wall surface, has been invoked less often. Texon (1957) suggested that the presence of lesions on the lateral walls of bifurcations could be explained by Bernoulli-type reductions in pressure. Such hypotheses have not gained acceptance, perhaps because the maximum dynamic pressure associated with the flow of blood is only around 10 mmHg (Caro et al., 1971) and hence point-to-point variation in pressure, scaled by the average value, will be much smaller than variation in WSS, similarly scaled.

Changes in blood pressure occur during each cardiac cycle and, because arteries are elastic, lead to cyclical changes in the dimensions of the wall. This strain my vary from site to site as a result of variation in wall stiffness or as a result of expansion being prevented by structures outside the wall. There is a segmental distribution of disease in the vertebral arteries, lesions occurring where the artery is free to expand, but not where it passes through the bone canal (Moossy, 1966; Solberg and Eggen, 1971). The internal carotid artery is protected from disease where it passes through the canal at the base of the skull (Glagov et al., 1988) and lesions are absent where coronary arteries pass under constraining “myocardial bridges” (Nakaura et al., 2014). Thubrikar et al. (1988) showed that lesion formation downstream of the renal artery ostium in cholesterol-fed rabbits can be prevented by restricting aortic expansion with an external cuff.

A fundamental problem with the evidence implicating arterial strain is that preventing a vessel from expanding will change not only strain but WSS as well—the time-average diameter will be smaller and hence WSS will be higher. The pattern of WSS may also be altered, if the geometry is complex. The same issue applies to *in vitro* studies examining effects of cyclic strain on cultured endothelium: when cells are stretched on a deformable membrane and there is static fluid above them, the stretch creates a relative motion with the fluid and thus generates fluid shear stresses. New techniques (e.g. Rowland et al., 2021) are required to look at more subtle, natural variations in strain, with the aim of determining whether it or WSS best explains the pattern of disease.

This summary understates the number and complexity of putative mechanical triggers. For example, there are hypotheses that depend on the temporal relation between WSS and strain (Dancu and Tarbell, 2007). What needs to be addressed is their relation to the change with age that occurs in the pattern of permeability and lesions. Broadly speaking there are two possibilities: 1) the distribution of mechanical stress is constant with age and it is the relation between mechanical stress and atherosclerosis that changes, or 2) the distribution of mechanical stress changes with age and the relation between mechanical stress and atherosclerosis remains constant.

(i) The relation between mechanical stress and atherosclerosis changes with age

A hypothetical example if this kind of mechanism would be that lesions develop at sites of high shear in immature aortas but at sites of low shear in mature aortas. That would require a change in the pathways between WSS and disease, such as in the relation between shear and permeability.

In the first study of age-related pathways (Forster and Weinberg, 1997), nitric oxide (NO) was chosen for examination because it can modify transport properties of blood vessel walls (Kubes and Granger, 1992; Yuan et al., 1992) and is influenced by age (Liu et al., 1992; Aliev et al., 1995), blood flow (Holtz et al., 1984; Rubanyi et al., 1986) and hyperlipidemia (Minor et al., 1990; Cooke et al., 1991). It was investigated in rabbit thoracic aortas; they were perfused with physiological buffer *in situ* to avoid indirect effects caused by the influence of NO on blood pressure, aortic flow, or interactions of blood cells with the vessel wall. Flow was steady. In this preparation, short-term uptake of albumin was greater downstream of branch points in immature aortas and upstream in mature aortas, as *in vivo*. That is itself interesting; it suggests that neither pattern is determined by unsteady components of luminal pressure or flow, interactions of blood cells with the vessel wall, or components of plasma, or that they are determined by these things via effects lasting longer than the 1.5 h that it took to conduct the surgery.

**FIGURE 6:**
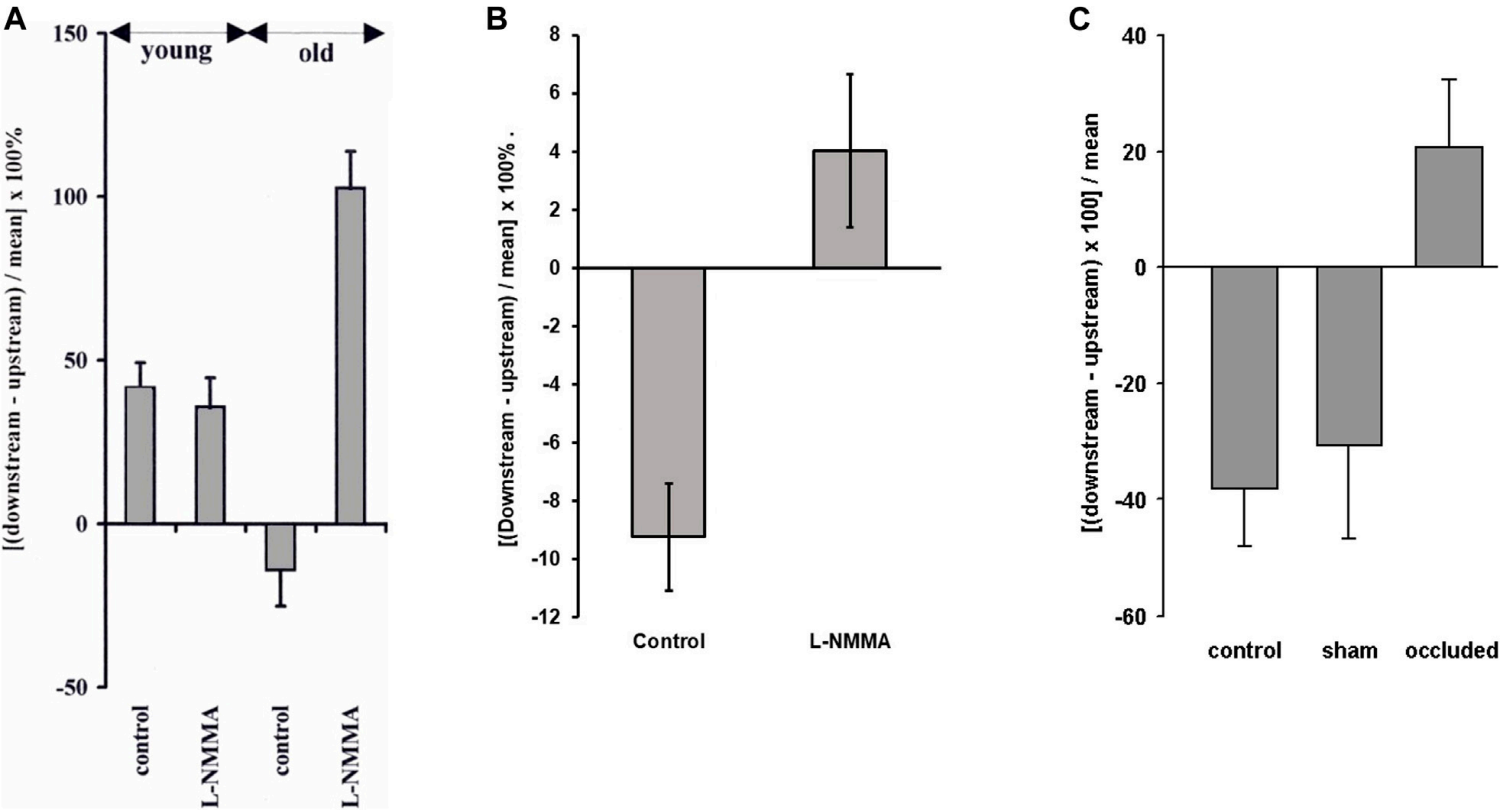
**(A)** The effect of the NO inhibitor L-NMMA on the pattern of albumin uptake upstream and downstream of intercostal branch ostia in the *in situ*-perfused rabbit aorta. Positive values indicate greater uptake downstream and negative values indicate greater uptake upstream, as in Figure 3A. L-NMMA reversed the normal mature pattern of uptake but had no effect on the pattern in immature rabbits (Forster and Weinberg, 1997). **(B)** A similar but smaller effect was seen in mature rabbits *in vivo* (Staughton and Weinberg, 2004b). **(C)** The same trend was seen in mature rabbits when the intercostal arteries were occluded in anaesthetised mature rabbits, whereas sham operation had no effect (Staughton et al., 2001).

When NO production was inhibited with L-*N* ^G^-monomethyl arginine (L-NMMA), starting 15–25 min before the administration of tracer, the mean of uptake upstream and downstream of intercostal branches in immature aortas increased by a factor of three but the pattern was unaffected. In mature aortas, mean uptake was unaffected but the pattern was reversed—uptake became greater downstream than upstream of branches and the difference was as large as that seen in the youngest immature rabbits (Figure 6A). This suggests a change in endogenous NO production (e.g. from basal production to flow-dependent production) and/or a change in the effects of NO on permeability with age.

Parenthetically, it is noted that EBD inhibits acetylcholine-stimulated relaxation of preconstricted aortic rings (Forster and Weinberg, 1997), the paradigmatic property associated with the NO pathway, and studies in which this intravital dye circulates for prolonged periods may therefore give results that do not truly reflect transport of native macromolecules *in vivo*.

A follow-up study examined the effects of L-NMMA on transport and cholesterol-induced lesions in rabbits *in vivo* (Staughton and Weinberg, 2004b). Only mature animals were used. The upstream pattern of short-term albumin uptake was seen in control animals, as expected. This pattern was abolished in the rabbits administered L-NMMA. The change was statistically significant, but uptake after L-NMMA was only slightly greater downstream of the branch than upstream and the difference between regions did not reach significance (Figure 6B). This is markedly different from the large effect seen in perfused vessels.

Lesions in the control cholesterol-fed group clustered around the lateral margins of the branch, and in areas extending upstream and downstream from these locations, as expected. The pattern was broadly similar in animals administered L-NMMA chronically during the trial, and pilot studies with a wide range of alternative NO inhibitors also failed to give the downstream pattern of lesions in mature animals.

The discrepancy between the *in vivo* and the *in situ*-perfused transport data could reflect the influence of NO on pressure, flow and blood cell-wall interactions, which are only present in the former. Alternatively, the discrepancy could represent a difference in transport mechanisms. Albumin has a size that gives approximately equal transport via “small pores” and “large pores” in capillary endothelium *in vivo* (Levick, 1991). These pores may represent intercellular junctions and vesicular routes, respectively (see below). Permeability to albumin was elevated up to five-fold in the perfused vessels compared to *in vivo*, most likely due to an increase in transport through intercellular junctions. The junctions may be more susceptible than vesicles to the influence of NO. On the other hand, the similarity of patterns of permeability *in vivo* and in perfused vessels argues that their determinants should be broadly similar. A simpler possibility is that the discrepancy may reflect an unknown technical issue; for example, NO synthesis could be harder to inhibit *in vivo*.

Further studies (Staughton et al., 2001) investigated whether there is a change with age in the sensitivity of the transport patterns to flow. This was achieved by tying off some intercostal arteries but not others in anaesthetised rabbits; fluorescently-labelled albumin was administered 20 min after the occlusion, and was allowed to circulate for 15 min before the aorta was fixed, sectioned and imaged. Uptake was greater upstream than downstream of control branches in mature animals, as *in vivo*, but the reverse pattern was seen at occluded branches, uptake being greater downstream, while mean uptake was unchanged (Figure 6C).

This is the same switch that was seen when NO was inhibited in perfused vessels (Forster and Weinberg, 1997), when hypercholesterolaemia (a known inhibitor of the NO pathway) was induced for 1 week (Sebkhi and Weinberg, 1994b), and when fixation was inadvertently delayed after death in *in vivo* experiments, which left vessels exposed to tracer in the absence of pressure and flow (Sebkhi and Weinberg, 1996). A partial effect in the same direction was seen when an NO synthase inhibitor was administered *in vivo* (Staughton and Weinberg, 2004b). All these data are consistent with an underlying downstream pattern being changed to an upstream pattern in mature animals when flow-dependent synthesis of NO occurs. Since mean uptake was not altered in these experiments, shear-dependent NO production—if it is responsible—increases transport upstream of the branch and decreases it downstream. The effect is rapidly reversible. (The latter point suggests that the presence of the normal mature pattern in vessels perfused with a steady flow of physiological buffier is *not* a lingering effect of the pulsatile flow of blood 1.5 h earlier.)

An unexpected result in the branch occlusion experiments (Staughton et al., 2001) was the absence of the downstream pattern in immature animals: uptake was equal in upstream and downstream regions. Furthermore, the pattern was unaffected by occluding the branches. An additional aberration was that mean uptake at branch points was three-fold higher in the immature animals than in the mature ones, contrary to the finding in perfused vessels and in conscious animals. These results may be indicative of some important mechanism underlying the immature pattern (for example, that the pattern is dependent on strain and therefore abolished by the hypotension arising from anaesthesia) or they may reflect some trivial experimental variable; the explanation is currently unclear.

A similar experiment that examined uptake of EBD over 20–30 min at the aorto-renal branch in a small sample of anaesthetised immature animals (Murphy, 1998) did find the normal pattern—greater uptake downstream of the branch than upstream—at control branches and showed that it was unaffected by partial ligation of the branch for 1.5 h. This suggests that the immature pattern does not depend on flow. Thus, different mechanisms appear to determine the uptake of albumin at immature branch points: not only is the pattern unaffected by inhibiting NO production but it also appears not to depend on flow, at least in the short term.

(ii) The distribution of mechanical stress changes with age

The data presented above strongly suggest that there is a change with age in the mechanism linking flow with patterns of transport near branches. There is no need to propose that the flow itself also changes with age. The first study (Al-Musawi et al., 2004) to test whether the pattern of flow is indeed constant exploited the relation between endothelial morphology and applied flow. Endothelial cell elongation and orientation, nuclear elongation and orientation, and the distribution of cytoskeletal F-actin all change when the direction and magnitude of the flow are modified *in vivo* (Levesque et al., 1986; Walpola et al., 1993) or *in vitro* (Dewey Jr et al., 1981; Levesque and Nerem, 1985); they have been widely used to assess WSS.

The study of Al-Musawi et al. found that endothelial cell nuclei were more elongated downstream than upstream of intercostal branch points in the aortas of immature rabbits. Lateral regions had intermediate elongations. In mature rabbits, by contrast, nuclear elongation was higher upstream than downstream; elongation in lateral regions was unchanged. This suggests that the distribution of WSS does reverse with age. The orientation of nuclei, putatively indicating the direction of near-wall flow, did not change.

**FIGURE 7:**
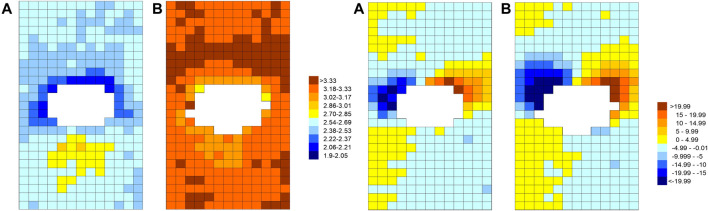
The pattern of endothelial nuclear elongation (left pair of maps) and alignment (right pair) around intercostal branch ostia (white) for immature (A) and mature (B) rabbits. Colour coding represents length-to-width ratios (left) or angles between the nuclear long axis and the aortic axis (right, with negative angles indicating the proximal end of the nucleus is displaced to the anatomical right). Maps show an *en face* view with mean flow from top to bottom (Bond et al., 2011).

This finding motivated a second study (Bond et al., 2011) in which elongation and orientation of endothelial nuclei were measured using automated methods. This permitted many more branches to be examined, and hence allowed the nuclear properties to be determined in smaller areas of the endothelium without incurring excessive variation. The resulting maps could be compared directly with maps of lesion prevalence. The study confirmed that the pattern of nuclear elongation, measured in rabbits aged 6–7 weeks and 11–22 months, reversed with age and that the pattern of nuclear orientation did not (Figure 7). The maps of elongation were remarkably similar to the maps of spontaneous disease in rabbits aged 4 weeks and 25–77 months (Figure 3B). Importantly, if cells and their nuclei elongate with increasing WSS, as commonly assumed, then this positive correlation suggests that both patterns of spontaneous rabbit lesions—and, by implication, the similar patterns of human disease—occur in regions of high rather than low WSS.

Additional findings of note, observed in the descending thoracic aorta as a whole, were: 1) a broad streak of high elongation along the dorsal surface, 2) many minor streaks of high elongation, also in the axial direction, bearing a strong resemblance to fatty streaks (which derive their name from this linear appearance), and 3) nuclear orientations that suggest counter-rotating, Dean-type vortices.

Thus, in addition to the evidence for a change in response to flow, described in the preceding section, these two studies suggest that the pattern of flow around branch points also changes with age. Only one of these mechanisms is required to account for the changing pattern of lesions. Biological systems are not selected by Occam’s razor, but the finding is unexpected—nothing about the flow profiles in Figure 1B suggests that it should occur—and it is therefore useful to examine assumptions underlying the nuclear elongation method.

First, most studies showing that elongation depends on WSS have examined cellular rather than nuclear morphology. Nevertheless, the study of Bond et al. (2011), just described, did include validation against a smaller series of measurements concerning elongation of the whole cell; a good correlation was obtained with nuclear elongation regardless of whether the cell outline was analysed manually or by using an automated technique based on machine learning (Iftikhar et al., 2011).

Second, there are other influences on cell elongation. For example, it is affected by flow pulsatility and reversal (Helmlinger et al., 1991) and by strain (Zhao et al., 1995). There also seems to be compensation for the increased WSS associated with smaller body size; endothelial cells know in some way the size of the body they are in (Weinberg and Ethier, 2007) and adjust their elongation accordingly. However, the effect of stretch is smaller than the effect of shear when both are applied at physiological levels (Zhao et al., 1995), the adjustment for body size should not influence local differences around branch ostia, and the influence of pulsatility and reversal do not contradict the view that the pattern of flow changes with age, only how it changes.

Third, the elongation of endothelial cells and their nuclei is a *response* to WSS. Evidence has been presented above that responses to shear can change with age. Hence it is feasible that endothelial elongation represents different flow features at different ages. The possibility that patterns of elongation reverse with age despite an unchanging flow pattern seems unlikely but cannot be ruled out *a priori*.

## Computational Studies of Flow

In the 1960s and early 1970s, when Fry and Caro et al. proposed their high- and low-shear hypotheses, the distribution of WSS had to be obtained by applying fundamental principles of fluid mechanics to simplified geometries or by using physical models. Over the years, models and associated measurement techniques became more advanced (e.g. Ku et al., 1985) and methods for characterising flow *in vivo* have also progressed (e.g. Riemer et al., 2021). However, current experimental methods still cannot reliably assess WSS around branch ostia.

The application of computational fluid dynamics (CFD), which occurred almost 40 years ago (Friedman and Ehrlich, 1984), was a big advance not only because it allows such assessment but also because it permits “experimental” manipulation of boundary conditions to determine their effect on the distribution of WSS; this is particularly valuable when attempting to understand effects of age.

An initial study with this aim (Kazakidi et al., 2009) determined influences of Reynolds number and side branch flow on the distribution of WSS around an ostium. The geometry was simplified by representing the branch as a cylindrical tube emerging perpendicularly from a flat surface. This assumption is justified where the parent vessel is much larger than the branch, as at an aorto-intercostal junction. The use of steady flow was justified by the desire to obtain time-average WSS and an assumption of quasi-steady flow. To examine effects of the branch alone, no attempt was made to incorporate the geometry of the aortic arch or its branches.

**FIGURE 8:**
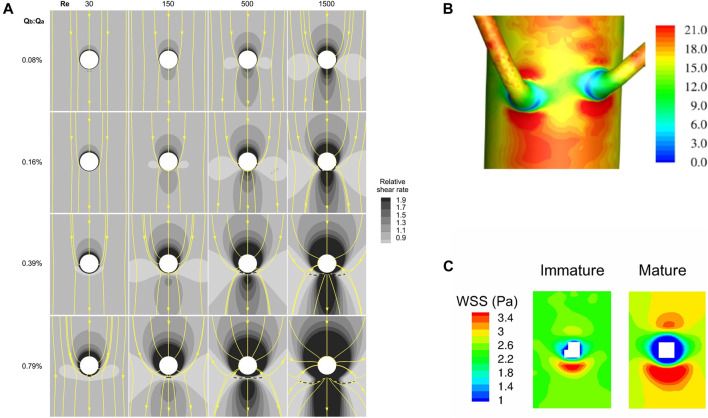
**(A)** Maps of the effect of Reynolds number and the ratio of branch to aortic flow rate on computed WSS around the ostium (white) of an idealised branch emerging from a flat plate, viewed *en face* with mean flow from top to bottom (Kazakidi et al., 2009). Surface streamlines are also shown. **(B)** Patterns of WSS (Pa) around a pair of aorto-intercostal junctions, computed for steady flow in a realistic representation of the rabbit anatomy and viewed from outside the vessel, with mean flow from top to bottom (Vincent et al., 2011). **(C)** Maps of WSS around intercostal ostia (white) computed for steady flow in realistic representations of an immature and a mature rabbit anatomy, viewed *en face* with mean flow from top to bottom (Peiffer et al., 2012).

The objective was to confirm that WSS is high downstream and low upstream of the branch, and to examine whether this pattern can be modified. The numerical simulations showed, however, that shear is highest upstream of the branch (Figure 8A). This arises because it is easiest for slow-moving fluid near the wall to change direction; contrary to the impression given by 2-D representations (Figure 1B), flow therefore enters the branch not only from near-wall regions that lie directly upstream of the ostium but also from other near-wall regions, spread over a wider region, and less from regions further away from the wall, even if they do lie directly upstream of the branch. The near-wall streamlines converging on the branch mouth indicate accelerating fluid and the higher velocity leads to higher WSS. This effect gets stronger as Reynolds number is raised, since the increased inertia makes it even harder for fluid away from the wall to enter the branch. It also gets stronger when side branch flow rate is increased, since more fluid needs to enter the branch. A patch of high shear stress upstream of the branch is visible in the physical models of Caro et al. (1971), although not discussed by them.

WSS was low lateral to the branch (Kazakidi et al., 2009) (Figure 8A). In this region, some flow turned to enter the branch while neighbouring elements continued down the parent vessel, leading to diverging surface streamlines. This effect increased with Reynolds number and side branch flow rate, as above. WSS was generally elevated downstream of the branch, presumably because the development of a new boundary layer overcame the effect of the diverging streamlines in this region, but at sufficiently high branch flow rates and low Reynolds numbers shear was actually lowest downstream.

This model therefore contradicts the assumptions on which both the high- and low-WSS hypotheses were predicated. Furthermore, no low- or high-shear region had a shape that particularly resembled any of the patterns of lesions described above. The closest resemblance was between the lateral pattern of lesions and the distribution of low shear at physiological Reynolds numbers (500 for the rabbit, 1,500 for people) and side-branch flow rates (0.16% or 0.39% of the aortic flow for each intercostal) (Figure 8A), but even it is not striking. Resemblances were not markedly improved by modelling branches in pairs or by using more realistic geometries for the inflow tract and flow divider.

A follow-on study (Kazakidi et al., 2011) introduced unsteady flow. Adding pulsatile but non-reversing side-branch flow whilst maintaining steady flow in the aorta produced only minor changes. However, if the side-branch flow reversed direction during part of the cycle, as may occur *in vivo*, WSS patterns were substantially disrupted: in the period of reverse flow, shear was reduced rather than elevated in a small region upstream of the branch. During the subsequent switch to forward side-branch flow, shear was reduced in substantial areas both upstream and downstream of the branch, and was high at the lateral margins, a pattern that was almost the reverse of the steady-flow case. A similar pattern could be induced during certain parts of the cycle in a case with reversing aortic flow and pulsatile but non-reversing side-branch flow, as could other patterns at other times. Despite this wide variety of instantaneous WSS patterns, once again none of them resembled lesion patterns particularly closely, and neither did the patterns of time average WSS or OSI.

Vincent et al. (2011) then examined WSS patterns at aorto-intercostal junctions in a realistic rabbit aortic geometry, obtained by micro-computed tomography of a cast that was created by infusing resin into the vessel *in situ* and at pressure, allowing it to set, and then dissolving away the tissue. This method gives spatial resolution on the order of 10 μm; disturbances in WSS caused by the ductus scar, for example, were easily visible. Viewing the branch mouth *en face*, high WSS was again apparent upstream and downstream of the ostium, with low WSS laterally. Values were generally higher downstream than upstream and the shapes of these patches were stretched circumferentially, or compressed axially, compared to the simpler models. When the simulations were viewed from outside the vessel (Figure 8B), a fine ring of much lower shear was observed around most of the circumference of the first part of the side branch. There was still no downstream triangle or upstream streak of low shear. The anatomy of the arch produced longitudinal streaks of low and high shear in the descending segment at Reynolds numbers relevant to the rabbit and human aorta, which may reflect Dean vortices formed in the arch. When these streaks intercepted a branch, their effects on WSS were additive with the patterns produced locally by the ostium.

In the final study focusing on high and low WSS, Peiffer et al. (2012) simulated steady flow in geometries derived from corrosion casts of young and mature rabbit aortas. The anatomy changed with age: aortic curvature and torsion did not differ significantly between the groups but there was more taper between the root and first intercostal artery in mature than immature rabbits. Increased taper led to greater persistence of arch-generated Dean vortices down the descending thoracic aorta, and this in turn led to higher WSS on the dorsal wall of the mature rabbits. Despite this, the pattern around intercostal branches remained constant with age—high WSS upstream and, even more so, downstream of the branch, and low WSS laterally—therefore disagreeing with the age-related pattern of endothelial nuclear elongation (Figure 8C). (The results were broadly similar to an earlier study by Buchanan et al. (1999) that mapped WSS at the rabbit aorta-coeliac junction.) In a subsequent statistical comparison (Peiffer et al., 2013b) of the WSS distributions with lesion patterns obtained in cholesterol-fed rabbits (Cremers et al., 2011), the correlation was of borderline significance for immature animals and not significant for mature ones. Even where borderline significance was obtained, the relation was positive; that is, high lesion frequencies were associated with high WSS.

## Potential Importance of Multidirectional Flow

The lack of resemblance between WSS and lesion patterns in these four CFD studies motivated a re-examination of the low WSS hypothesis. With the aim of assessing the strength of the spatial correlation in previous studies, a systematic review (Peiffer et al., 2013c) examined all papers identified with the search terms atherosclerosis, shear and computational fluid dynamics, or synonyms of them. Twenty-seven papers remained of the original 406 after applying various pre-defined inclusion and exclusion criteria (e.g. the geometry had to be anatomically realistic, but not include severe disease that would itself have affected flow). Of these studies, 22 stated that their results agreed with the low WSS theory and only 5 (<20%), all examining thickening of human arteries, did not. That appeared to be strong evidence for the low WSS hypothesis.

However, a different picture emerged when the studies were subdivided according to the degree of data reduction and level of quantification they had employed to compare the patterns of WSS and disease. Studies that used descriptive analysis, visual comparison, simple thresholding or preselected areas unanimously supported the hypothesis. Studies that were quantitative but used substantial data reduction (circumferential or axial averaging) were evenly divided for and against the hypothesis. And all studies that conducted a quantitative, point-by-point comparison rejected the hypothesis. The fact that the most rigorous studies were the ones that rejected the hypothesis is even more striking when it is recognised that standard statistical techniques tend to over-estimate significance, due to problems with autocorrelation (Peiffer et al., 2013b; Rowland et al., 2015).

Although discussion about mechanical factors in atherogenesis has been dominated by consideration of high, low and oscillatory WSS, many other triggers have been proposed (see above). Some are physically unrealistic and no longer under active investigation, but others are based on comparison between lesion patterns and maps derived from numerical simulations of flow. Multidirectional WSS, occurring where near-wall flow changes direction over the cardiac cycle, has been the subject of several recent studies, and is discussed further here.

One metric defining multidirectional shear is the transverse WSS (transWSS) (Peiffer et al., 2013a):

$$\mathrm{transWSS}=\frac{1}{T}\int_{0}^{T}\left|{\vec{\tau }}_{\omega }\cdot \left(\vec{n}\times \frac{\int_{0}^{T}{\vec{\tau }}_{\omega }dt}{\left|\int_{0}^{T}{\vec{\tau }}_{\omega }dt\right|}\right)\right|dt$$

$$\mathrm{transWSS}=\frac{1}{T}\int_{0}^{T}\left|{\vec{\tau }}_{\omega }\cdot \left(\vec{n}\times \frac{{\vec{\tau }}_{mean}}{\left|{\vec{\tau }}_{mean}\right|}\right)\right|dt$$

where $\vec{n}$ represents the normal to the arterial surface.

It averages over the cardiac cycle those components of the instantaneous WSS vectors that are perpendicular to the mean WSS vector.

Subsequent, related metrics include the ratio of the magnitude of circumferential and axial WSS projections (Morbiducci et al., 2015) or the tangent and binormal directions (Arzani and Shadden, 2016), and the anisotropy ratio (Vamsi Krishna et al., 2020). There is also some relation to the earlier Directional Oscillatory Shear Index (DOSI) (Chakraborty et al., 2012), which captures the relative oscillatory character of flow along orthogonal axes, and the aneurysm formation indicator (AFI) (Mantha et al., 2006), which is the cosine of the angle between the instantaneous and mean WSS vectors and hence does not consider WSS magnitude; it gives more weight to reverse flow than to flow perpendicular to the mean vector.

The Cross Flow Index (CFI) (Mohamied et al., 2017) is defined in a similar way to the transWSS but, like the AFI, does not take into account the magnitude of the instantaneous vectors. The minimised transverse WSS (transWSSmin) (Ghim et al., 2017) is also defined in a similar way to the transWSS but uses as the reference not the mean WSS vector but the orientation that minimises the calculated transWSS. It is not clear that *in vivo* experiments will ever be able to discriminate between effects of these different metrics. The focus here is on transWSS and transWSSmin because they are defined with regard to endothelial biology: they describe the WSS occurring across the long axis of endothelial cells that are aligned with the mean WSS vector or that align so as to minimise such transverse WSS, respectively.

**FIGURE 9:**
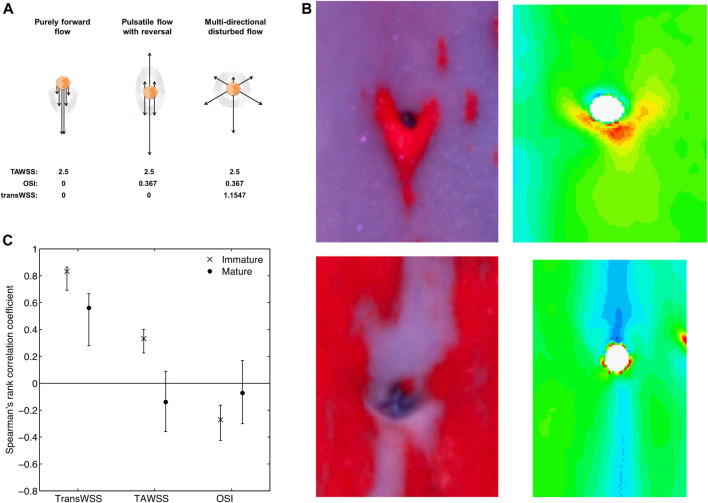
**(A)** Sketch showing that the same time average WSS (TAWSS) and OSI can occur for markedly different types of flow, therefore justifying the need for the transWSS metric capturing the directionality of flow. Dark arrows represent instantaneous flow vectors and the large grey arrow represents time throughout one cycle (Peiffer et al., 2013a; Mohamied et al., 2015). **(B)** Examples of diet-induced lesions (left) and computed transWSS (right) around intercostal branch ostia in the aortas of immature (top) and mature (bottom) rabbits, viewed *en face* with mean flow from top to bottom. (Peiffer et al., 2013a). **(C)** Coefficients (mean and confidence interval) for the correlation between patterns of lesions and patterns of each of the three WSS metrics, in immature and mature rabbits. Correlations are significant if the confidence interval does not include zero (Mohamied et al., 2015).

A metric characterising transverse flow is required because the OSI is increased by instantaneous WSS vectors that are aligned with the axis of the mean WSS vector but point in the opposite direction, as well as by vectors that have components at right angles to the mean vector. Hence the same value of OSI can be obtained for flows that are entirely different in character (Figure 9A). Uniaxial but reversing flow must occur in many straight segments of conduit arteries and should probably not be regarded as atherogenic. True multidirectionality, on the other hand, would be expected at bends and branches. It is not restricted to stagnation points, contrary to the hypothesis of McMillan (1985a,b).

The fundamental pattern of transWSS produced by a branch was investigated using the highly idealised model of a cylindrical tube emerging from a flat surface, described above (Peiffer et al., 2013a). Under pulsatile non-reversing or reversing side-branch flow, or with pulsatile aortic and branch flow, transWSS was always elevated in a four-lobed “butterfly” pattern, with high values at the proximal left, proximal right, distal left, and distal right regions around the ostium. The pattern was strikingly different from those for time average WSS, the OSI or the relative residence time.

Long, axial streaks of high transWSS were seen in anatomically realistic models of the rabbit aorta, derived from corrosion casts (Peiffer et al., 2013a). They occurred at different anatomical locations from streaks of low WSS, high OSI and high residence time, all of which coincided with one another. The streaks of high transWSS lay closer to the ostia of the intercostal branches. (There was a strong resemblance to the streaks of disease observed in mature rabbits by Cremers et al. (2011)). Of critical importance, the interaction of these streaks with the branch-dependent patterns of transWSS was not simply additive, contrary to the behaviour of WSS discussed above: the pattern around branches was changed. In cases where the ostium lay within a streak, a downstream arrowhead of high transWSS was seen, whereas when the ostium lay at the edge of a streak, high transWSS was seen at the lateral margins of the ostium, and in regions extending upstream and downstream from these locations. The patterns bore a striking resemblance to the age-related patterns of lesions seen in cholesterol-fed rabbits (Figure 9B).

The pattern of transWSS around branches could change with age if the streaks of high transWSS move relative to the branches, perhaps as a result of changes in aortic geometry. Patterns of transWSS were therefore computed for geometries derived from the corrosion casts of young and mature rabbit aortas (Mohamied et al., 2015), even though time average WSS itself was already known not to change. The results for transWSS, as well as for time average WSS and OSI, were compared with the earlier study of lesion patterns in cholesterol-fed rabbits (Cremers et al., 2011) using more powerful, “bootstrapping” statistical methods.

TransWSS correlated significantly with lesion prevalence in mature animals, but time average WSS and the OSI did not (Mohamied et al., 2015). TransWSS also correlated significantly with lesion prevalence in immature animals and, although the other two metrics also did so (positively for time average WSS and negatively for the OSI), the correlation with transWSS was significantly stronger. Indeed, the correlation coefficients for transWSS were approximately three times those observed for the other metrics at both ages, exceeding 0.8 for immature rabbits. No significant correlations supported the low, oscillatory WSS theory. Thus the data are consistent with lesions being triggered by high transWSS in both immature and mature aortas, and with the pattern of this metric changing with age (Figure 9C).

Large-scale features of the pattern of transWSS in the thoracic aorta appear to be generated during the acceleration, peak systolic and deceleration phases of the cardiac cycle; the reverse flow and subsequent diastolic phases are short or characterised by WSS magnitudes that are close to zero, respectively, and hence do not make a significant contribution (Mohamied et al., 2017). The features are insensitive to the inflow waveform but sensitive to geometry. The curvature of the arch and descending aorta produce vortical structures that are responsible for the streaks of high transWSS; streaks in the descending segment can be decoupled from those in the arch and thus appear to depend at least in part on local curvature. Non-planarity is responsible for asymmetries in the streaks and taper determines their persistence down the aorta, as with time average WSS (Mohamied et al., 2017).

The mechanisms by which the pattern of transWSS around branches is generated, and by which it changes with age, remain under investigation but, as already noted, changes in the location of the large-scale streaks may be responsible and these could in turn be determined by alterations of geometry. Changes with age in the taper of the rabbit aorta have been discussed above. Human aortas are known to “unfold” with age—the arch gets longer and its radius of curvature increases (Redheuil et al., 2011; Rylski et al., 2014)—whilst the angle of kyphosis, indicating curvature of the upper back, increases (Fon et al., 1980). Note that the parallel change with age in rabbit and human lesion patterns does not require that geometric changes are the same, only that vortices are shifted in the same way. Note also that the sensitivity of branch patterns to geometric features means that the results of CFD simulations could depend on subtleties such as the posture of the rabbit during the aortic casting process (see below); this may explain why changes with age in the average transWSS patterns are not as dramatic as changes with age in lesion patterns (Mohamied et al., 2015), or as the examples shown in Figure 9B.

Interactions between branch and large-scale patterns of transWSS may help answer another long-standing issue concerning the distribution of disease, which is that the influence of branches on lipid deposition appears to propagate further from the ostium than any conceivable influence branches have on flow (Weinberg, 2002). Previously, this anomaly has been attributed to the migration of activated endothelial cells towards the heart (Weinberg, 2002) but it may more plausibly be explained by the merging of branch-dependent and vortex-dependent regions of high transWSS.

### Conclusion

CFD simulations were run with the aim of resolving whether the pattern of WSS around intercostal branch ostia changes with age in a way that can explain the age-related pattern of lesions, as suggested by patterns of nuclear elongation, or whether the pattern of WSS is static and therefore a change in the response to WSS is a more likely explanation, as suggested by experiments that altered NO synthesis and flow. The simulations showed that patterns of WSS did not change markedly between immature and mature rabbits. However, they did not support the idea that a change in response was responsible either, because the patterns were not those expected from early theoretical or physical models and did not strongly resemble the pattern of lesions at any age. The CFD simulations introduced a third possibility: they showed that the pattern of a new metric—the transWSS, capturing multidirectional WSS—changed with age and showed a much stronger resemblance to the pattern of lesions at both ages.

The discrepancy between the pattern of endothelial nuclear elongation and the pattern of WSS derived from CFD was striking. Not only did the former change with age whilst the latter did not, but the pattern of elongation did not strongly resemble computations of the pattern of WSS on which it is supposed to depend. Assumptions and simplifications are always involved when computing flow but the parametric studies suggest that only a fundamental error in specifying the side-branch flow waveform would have a large enough influence to annul the discrepancy. Doppler ultrasound data of Sloop et al. (1998) are consistent with an age-related change in waveform, reverse flow disappearing in people between the ages of 30 and 40 years. However, children were not examined. Further investigation is required.

The two data sets could be resolved if endothelial cells elongated in response to high transWSS rather than to high WSS. However, several studies which applied multidirectional flow to endothelial cells *in vitro* have shown the contrary (Dardik et al., 2005; Chakraborty et al., 2012). This topic also requires further investigation. For example, the same elevated value of transWSS can be produced by high WSS with small changes in flow direction, or by low WSS with large changes in direction. It is conceivable that different combinations occur *in vivo* and *in vitro* and have different effects on elongation.

The emergence of a possible role for transWSS introduces yet another factor into the interpretation of the experimental studies that appeared to show a change with age in the response to flow. Inhibiting NO synthesis had a dramatic effect on the mature but not the immature pattern of transport, but the data were obtained in aortas perfused *in situ* with a steady flow of buffer (Forster and Weinberg, 1997). There is zero transWSS everywhere when flow does not vary cyclically. Under such conditions, other fluid mechanical stresses may assume an importance in controlling transport into the wall that they do not have when significant transWSS is present. The fact that transport was measured only in regions directly upstream and downstream of branches could have hidden changes in pattern produced by such a shift.

Limitations imposed by measuring only directly upstream and downstream may also be relevant to the studies where NO was inhibited by giving L-NMMA to mature rabbits *in vivo* (Staughton and Weinberg, 2004b); the normal pattern of uptake—transport greater upstream than downstream—was abolished, but it was not dramatically reversed, as it was in the perfused vessels. This issue was reinvestigated in the studies (Bailey et al., 2015) where transport around branches was mapped *en face* by confocal microscopy. Some rabbits were administered high dose heparin for 1 h, which is known to inhibit the synthesis of NO (VanTeeffelen et al., 2007). Only very subtle changes were seen in the map of albumin uptake for 16–22-month-old animals but, although they were visually hard to discern, they were sufficient to change the pattern directly upstream and downstream of the branch from being ≈8% greater upstream to ≈3% greater downstream; these numbers are identical to the earlier study and, as before, the change was significant. The implication is that measurements restricted to the branch centreline may give an unrepresentative view and that inhibiting NO may do very little in conditions where transWSS is present. The same argument could also apply to the effect of side-branch ligation in mature animals, which appeared to reverse the normal pattern of transport (Staughton et al., 2001): the size of the effect may similarly have been incorrectly estimated.

A final point arising from the CFD studies is that the putative effects of aortic curvature on the large-scale distribution of transWSS, and hence on its pattern around branches, mean that the posture of the animal (or human subject) may be of importance. The *in situ* perfusion experiments, the side-branch ligation experiments and the casting of the aorta were done with the rabbits supine, whereas rabbits were upright during tracer administration in the *in vivo* experiments, including when NO was inhibited. Long-term responses, such as those responsible for endothelial nuclear elongation or lesion development, involve integration over different postures.

## Further Investigation of Mechanisms Relating Flow to Permeability

In the following, it is assumed that the pattern of flow changes with age. That would be consistent with an age-*independent* set of mechanisms linking flow, via transport, to disease. Such mechanisms have been sought *in vivo*, *in silico* and—for more molecular mechanisms—*in vitro*.

(i) *In vivo*

The two-pore theory of transendothelial transport was discussed above. The normal intercellular junction cannot be the large pore because it is too narrow to allow passage of particles such as LDL (diameter 23 nm). However, the cell turnover leaky junction hypothesis of Weinbaum et al. (1985) proposes that the width of intercellular junctions temporarily increases when endothelial cells divide or die, and that large particles might then cross by this route. Consistent with this hypothesis, “hotspots” of high permeability have been observed for a range of macromolecular tracers (reviewed by Chooi et al., 2016). They are associated with enhanced entry of LDL and often, although not exclusively, with mitosis or apoptosis (Stemerman et al., 1986; Lin et al., 1988; Lin et al., 1989; Lin et al., 1990; Truskey et al., 1992; Malinauskas et al., 1995); in rabbits weighing <3 kg, they occur at high frequency downstream of aortic side branches (Barakat et al., 1992; Herrmann et al., 1994). Mitosis and apoptosis are both thought to depend on blood flow.

One study (Chooi et al., 2016) has investigated whether the pattern of hotspots and mitosis changes with age around rabbit intercostal branch ostia. EBD pre-mixed with albumin was used as the tracer; it was allowed to circulate for only 10 min in order to limit any inhibition of the NO pathway. Hotspots of high uptake, as well as normal uptake, were quantified by *en face* confocal microscopy of tracer fluorescence, and mitosis occurring over several days was mapped by adding bromodeoxyuridine to drinking water and then staining it in endothelium with a fluorescent antibody. These methods should give better quantification and higher sensitivity than was possible in some earlier studies.

The main finding was that the pattern of hotspots switched with age. Hotspots were more frequent and more leaky downstream of branches in immature animals, and in longitudinal streaks at the sides of branches in mature animals. The patterns resemble those seen for disease and for uptake of other tracers except that areas immediately lateral to the branches had few hotspots. This may have resulted from a relative paucity of elastin and collagen in such regions, since EBD binds to these proteins on entering the wall.

Despite the detection of two patterns of hotspots, closer examination of the data did not provide much support for the cell turnover leaky junction hypothesis. First, there was no discernible pattern of mitosis and no spatial correlation between the number of mitosing cells and any hotspot metric. Second, hotspots accounted for only 5% of total uptake. Third, the non-hotspot uptake showed exactly the same patterns, and so did uptake through only the largest hotspots. These observations suggest that there is a continuum of local permeability values, and that the majority of uptake does not reflect the occurrence of an aberrant event. (Hotspots may have been caused by apoptosis, which was not assayed, and hotspots might have played a more significant role if a larger tracer had been used.) The relation between mitosis or apoptosis and shear stress is also not straightforward. The paper most often cited to show that DNA synthesis depends on flow—Davies et al. (1986)—showed an effect of turbulence, but there was actually no difference between static, low shear and high shear conditions. Furthermore, shear decreases rather than increases apoptosis (Dimmeler et al., 1996).

(ii) *In silico*

Concentration polarisation at the luminal surface of the arterial wall has been investigated by both computational and experimental methods. However, the experimental studies have been conducted under conditions where concentration polarisation is expected to have been unphysiologically high (reviewed in Vincent and Weinberg, 2014), so the focus here is on *in silico* work.

Concentration polarisation is expected because the arterial wall is two orders of magnitude more permeable to water than to large particles such as LDL. It will occur at the luminal surface if the endothelium provides the major barrier to LDL entry. A number of processes would act to reduce such LDL accumulation. First, LDL does cross the endothelium into the wall at a measurable rate; this process will accelerate as the concentration of LDL increases, unless it occurs by a saturable mechanism. Second, LDL can diffuse away from the endothelium, back into the bulk of the plasma; this is the classical effect that limits polarisation. Third, the flow of blood within the lumen could advect the LDL away from the wall. Because it would influence the near-wall concentration of LDL, and hence the transport of LDL into the wall, this mechanism provides a potential link between near-wall blood flow and uptake by the wall of large molecules (Colton et al., 1972; Keller, 1974).

Considering the case of flow down an infinitely long, straight artery in which concentration polarisation has already occurred, luminal flow would have no influence on the concentration of LDL at the wall sufficiently far from the entrance. The fluid would be shifted axially at each location, by an amount that depended on the distance from the wall and on the global flow rate, but that would not affect the concentration profile within the boundary layer, which varies only in the radial direction. An effect of blood flow rate and wall shear stress on surface LDL concentration was seen in a straight artery in the computations of Wada and Karino (1999) because the LDL concentration was assumed to be uniform across the diameter at the entrance of the artery, and the segment was only 20 diameters long; hence, the concentration profile in the boundary layer was not the same at all lengthwise locations.

A second study by the same authors (Wada and Karino, 2002) examined flow in a geometry with a double bend, where flow recirculation occurred. In this case, the presence of blood flow with radial components would be expected to modify the concentration boundary layer even in the absence of entrance effects. Local variation in the surface concentration of LDL was seen in the simulation, although the proportion due to entrance effects was not established.

It is interesting to speculate that a branch could also modify near-wall concentrations, if it were located sufficiently far from the inlet that momentum and concentration boundary layers had developed. Considering, as a simplified example, the 2-D model described in Figure 1B, in which the near-wall blood upstream of the branch leaves the aorta, and the wall downstream of the branch is exposed to blood that had been closer to the aortic centreline, a lower near-wall LDL concentration would be expected downstream than upstream of the branch.

How big might such effects be? Wada and Karino (2002) obtained surface LDL concentrations 35% above the bulk concentration in their model of a double bend, albeit at Reynolds numbers below those appropriate for the human or even rabbit aorta. The model assumed that the wall is uniformly permeable to water, and that there is a sharp boundary between the blood and the wall. Neither assumption is correct. Fluid flow across the endothelium occurs principally through intercellular junctions, which occupy ∼0.1% of the surface area. Fluid fluxes need to be confined to these areas in computational models, and to be increased by three orders of magnitude to maintain the same global hydraulic conductance. The luminal surface of the endothelium is covered with a coat of glycoproteins, proteoglycans and adsorbed plasma macromolecules; the thickness of this glycocalyx layer is debated, but current estimates are in the micron range (van den Berg et al., 2003). The layer has a higher resistance to diffusion than blood and shields the wall from convection in the blood phase. The first effect increases concentration polarisation and shear dependence, but only by small amounts at physiological parameter values (Vincent et al., 2009). The second effect amplifies the influence of the first on concentration polarisation because of the decrease in LDL diffusion (Vincent et al., 2010). On the other hand, it reduces the influence of luminal blood flow (Vincent et al., 2010). Overall, the predicted entry of LDL into the wall is essentially independent of the applied shear.

(iii) *In vitro*

Molecular mechanisms linking flow and endothelial permeability have been extensively investigated *in vitro*. However, there are four common issues with such studies.

The first is that they usually investigate responses to acute changes in flow—most often, a step change from zero flow to some steady level. This is convenient experimentally; many methods for applying shear stress to endothelial cells do not permit long-term culture. But the responses may be different—in fact, opposite—to those caused by chronic exposure to flow, and the mechanisms also differ. In a study by Warboys et al. (2010), exposure of endothelium to shear for 1 h increased permeability to albumin whereas exposure for 1 week decreased it, compared to static culture. The effect of chronic but not acute shear was reversed by inhibiting phosphatidylinositol-3-OH kinase, NO synthesis or soluble guanylyl cyclase.

Chronic *in vitro* effects are likely to prove more relevant than acute ones to endothelial behaviour *in vivo*. It could be argued that the transient effects are relevant to the inability of endothelial cells ever to adjust to constantly changing “disturbed flow,” but a control involving chronic shear would still be required in order to determine effects of such flow.

If only studies of chronic shear are admissible, then the field is a small one. To make matters worse, a second issue is that most investigations have focused on unidirectional flow or, at best, flow that is oscillatory or pulsatile along one axis. If transWSS elevates permeability, then studies of uniaxial flow are insufficient. Unfortunately, it is difficult to apply multidirectional flows *in vitro*. The standard parallel-plate flow chamber has been adapted for this purpose. Kataoka et al. (1998) added inlet and outlet ports on the sides of the chamber, allowing flow to be switched between conventional and orthogonal directions, whilst Wang et al. (2012) grew endothelial cells on a glass slide within the chamber that could be manually rotated by any angle. The flow is predictable and controllable with both methods but throughput is low, chronic exposure is challenging and the frequency of directional change is many orders of magnitude lower than *in vivo*.

An alternative is the orbital shaker or swirling well method, in which endothelial cells are grown in conventional dishes or multi-well plates placed on a horizontal platform that translates in a circular orbit in the plane of the platform. The translation induces a wave that swirls around the well, producing multidirectional flow in the centre and uniaxial flow at the edge. The method (reviewed by Warboys et al., 2019) is low cost, high throughput, and permits chronic application of multidirectional flow at an appropriate frequency. The flow can be altered by changing the orbital radius and velocity, the dish radius and the depth of medium, but it is difficult to separate the effects of different shear metrics, several of which change with increasing radial distance from the centre. Furthermore, although different flow regimes have been identified and approximate analytical methods devised (Alpresa et al., 2018a; 2018b), accurate description of the shear acting on the base of the well requires CFD models that include the orbital forcing, the free surface of the gravitational wave (Berson et al., 2008) and, in some cases, the surface tension (Arshad et al., 2022).

Over twenty studies have used the method to determine effects of shear on endothelial cells; they demonstrated more homeostatic behaviour in endothelial cells located near the edge (uniaxial flow) and more pathogenic behaviour towards the centre (multidirectional flow) (Warboys et al., 2019). An interesting recent observation (Arshad et al., 2021) is that endothelial cells can align so as to minimise the transWSS they experience; an observation of this type could not have been made with conventional devices producing unidirectional or uniaxial flows. It suggests that the effects of transverse flow on endothelial cells are sufficiently harmful that the cells have evolved mechanisms for minimising it. The adverse effect may be as simple as the higher shear stresses, or shear stress gradients, experienced by regions of the cell membrane that cover the nucleus and protrude into the lumen (Barbee et al., 1995; Hazel and Pedley, 2000; Yamaguchi et al., 2000), or they may involve more complex events (Wang et al., 2013). In either case, the behaviour justifies using the transWSSmin metric, described above.

A third issue is that endothelial permeability to albumin is two orders of magnitude higher in culture than *in vivo* (Albelda et al., 1988). A parallel phenomenon is seen in organs or vessels that are perfused with buffer rather than blood. Many groups have searched for ways to reduce this hyperpermeability; recent studies have identified the lack of sphingosine-1-phosphate (S1P) as a key issue. In blood, S1P is chiefly carried by erythrocytes and platelets; adding it to cell culture and to perfused organs or vessels dramatically reduces paracellular permeability (Schaphorst et al., 2003; Curry et al., 2012; Warboys et al., 2012; Zhang et al., 2016). In its absence, factors controlling permeability may be misidentified.

The fourth issue is that studies of mechanisms linking flow and transport need to examine the relevant transport pathway. If, for example, it is LDL transport that is of interest, then examining effects of flow on normal intercellular junctions may have little value. Unfortunately, routes are difficult to determine because transit times are short, and because tracers that cross the endothelium via junctions can move laterally to appear under the cell body and those that cross via vesicles can similarly appear under junctions. For LDL transport, the consensus view has swung back and forth between junctions and vesicles.

A significant breakthrough was the development by Dubrovskyi et al. (2013) of a method in which endothelial cells are grown on biotinylated gelatin and a tracer comprising avidin (approx. 66–69 kDa, similar to albumin) labelled with fluorescein isothiocyanate (FITC) is added to the medium above the cells. When this tracer crosses the endothelium, it immediately binds to the substrate. Its distribution, imaged by fluorescence microscopy, can be compared with overlying structures to identify the transport route. FITC-avidin was unequivocally found under intercellular junctions.

**FIGURE 10:**
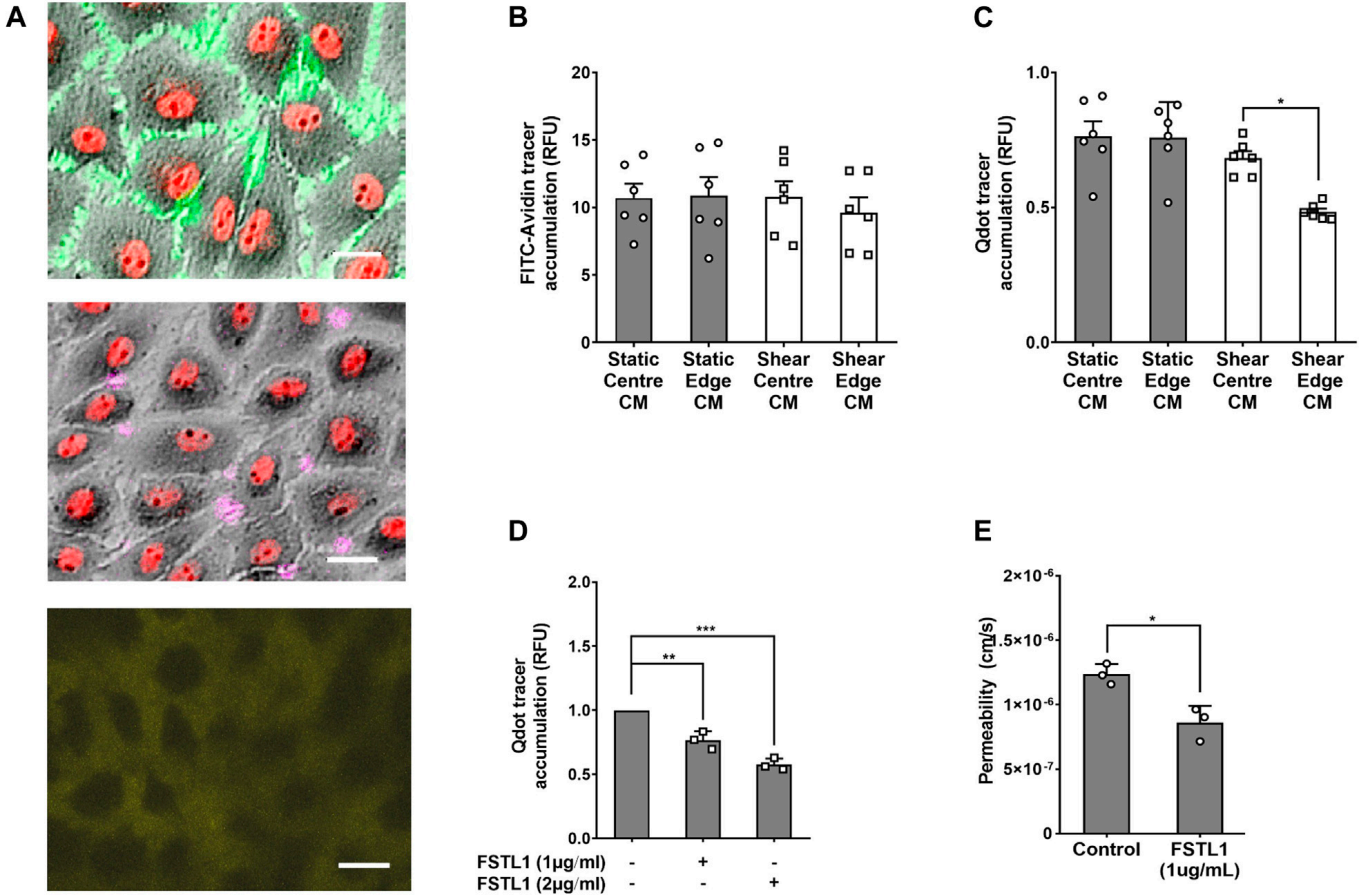
**(A)** *En face* view of the uptake of avidin based tracers that have crossed cultured endothelium and then immediately bound to the biotin-labelled substrate. From top to bottom: albumin-, HDL- and LDL-sized tracers comprising avidin labelled with FITC (green), phycobiliprotein (purple) and quantum dots (yellow), respectively. Endothelial cells are visible in phase contrast and their nuclei are stained red (DRAQ-5) in the top two images. Bar = 10 µm (top and middle) or 20 µm (bottom) (Ghim et al., 2017). **(B,C)** Conditioned medium (CM) from cells at the centre or edge of swirled or static wells had no effect on the permeability of target cultured endothelium to FITC-avidin, but CM from cells swirled at the edge reduced permeability to quantum dot-labelled avidin. RFU = relative fluorescence units (Ghim et al., 2021b) **(D,E)** Exogenous recombinant FSTL1 reduced permeability of cultured endothelium to quantum dot-labelled avidin and to LDL (Ghim et al., 2021b).

Ghim et al. (2017) extended this concept. Using other fluorophores can give larger tracers whilst retaining the essential biotin-binding property. Labelling avidin with a phycobiliprotein gives a tracer around the size of high density lipoprotein (HDL) whilst labelling it with a quantum dot (Qdot800) gives a tracer the size of LDL. Three pathways were observed. The albumin-sized tracer crossed the endothelium via junctions between two or more cells. (The fraction crossing where two rather than three or more cells meet depends slightly on experimental conditions; human endothelial cells cultured in appropriate medium form a tighter barrier than porcine aortic endothelial cells cultured in DMEM, and less FITC-avidin is then transported through bicellular junctions (Ghim et al., 2021a).) The HDL-sized tracer crossed *only* where three or more cells came together. And, despite the elevated paracellular transport expected *in vitro*, the LDL-sized tracer crossed only through the cells (Figure 10A).

Accumulation of the LDL-sized tracer under the cells was reduced in the nuclear region and was not enhanced near cell junctions even after prolonged incubation (Ghim et al., 2017). Furthermore, Ghim et al. (2021b) subsequently showed that the subendothelial accumulation of the tracer, but not of FITC-avidin, was reduced by Dynasore, which is an inhibitor of the fission of clathrin-coated vesicles from the plasma membrane (Macia et al., 2006) and also of fluid-phase endocytosis and micropinocytosis (Park et al., 2013). These observations are consistent with transport by a vesicular pathway of some type. The three pathways identified by Ghim et al. (2017) are different from the three—normal junctions, transiently widened junctions and vesicles—deduced by Cancel et al. (2007); for LDL transport under convective conditions, they determined that leaky junctions were the dominant route.

Strong evidence for vesicular transport of LDL has also arisen from studies identifying receptors—scavenger receptor class B type 1 (SR-B1) (Armstrong et al., 2015; Ghaffari et al., 2018; Huang et al., 2019) and activin receptor-like kinase 1 (ALK1) (Kraehling et al., 2016)—that mediate its passage in coronary artery and aortic endothelia, and from studies showing reduced LDL transport in caveolin 1 deficient mice (Ramírez et al., 2019). (Receptor mediated transcytosis of albumin has also been proposed (Schnitzer, 1992)). The influence of receptors may have been over-estimated in some of these investigations. For example, the concentration of labelled LDL was often far below physiological levels, which would have favoured high-affinity routes. It is unlikely that a receptor exists for quantum dots, so fluid phase transcytosis seems the most plausible mechanism at least for this tracer, and perhaps also for LDL itself (Vasile et al., 1983).

The swirling well method was used to investigate effects of different types of flow on transendothelial transport of the three avidin-based tracers. The choice of culture plate, medium depth and orbital parameters gave a large difference in the directionality of WSS from the centre to the edge of the well, but a negligible change in time average WSS, according to numerical simulations that incorporated surface tension (Arshad et al., 2022). Transport of the small and medium-sized macromolecular tracers through intercellular junctions was increased in the centre of the well (multidirectional flow) and decreased at its edge (uniaxial flow) compared to static controls (Ghim et al., 2017). However, transcellular transport of the LDL-sized particle was uniformly lowered across the well.

## Proposal of a Secreted Mediator

Why there should be a uniform change in LDL transport despite spatial variation in the type of flow? One possibility is that a soluble mediator was being secreted by cells in one region, exposed to one type of flow, and that it became well-mixed in the medium, thus affecting all regions equally, overcoming any effects of local flow on LDL permeability (Ghim et al., 2017). If correct, this would mean that the directionality of flow affects transport of both small and large macromolecules, but through different mechanisms; that might explain why transport patterns for albumin (Weinberg, 1988) and LDL (Schwenke and Carew, 1988) are similar *in vivo* even though transport might be occurring through different pathways.

To test the concept that secreted mediators could obscure the true relation between flow and endothelial phenotype, methods were developed to passivate parts of the base of the swirling well so that cells would grow only at the edge or only at the centre, even in chronic experiments (Ghim et al., 2018; Pang et al., 2021a; Ghim et al., 2021b). When the whole well was seeded with cells and swirled, there was no difference in tumor necrosis factor α (TNF-α)-induced intercellular adhesion molecule 1 (ICAM-1) or vascular cell adhesion protein 1 (VCAM-1) expression between the centre and the edge (Ghim et al., 2018). When only the centre or only the edge was seeded, expression was unchanged at the edge but elevated in the centre (Ghim et al., 2018). This demonstrates that an anti-inflammatory mediator is released from cells at the edge (uniaxial flow). When the whole well is seeded or when only the edge is seeded, the mediator limits adhesion molecule expression by all the cells. When cells are not present at the edge, there is increased expression by cells in the centre.

The method can be extended by collecting medium from fully seeded wells, or from wells where cells grow only at the centre or edge, and then applying the conditioned medium to target cells. This technique was used to unmask the true relation between flow and LDL transport across cultured endothelium (Ghim et al., 2021b). Medium conditioned by cells sheared at the edge of the well (uniaxial flow) reduced transcellular transport of the LDL-sized tracer in target cells but did not affect transport of the smaller, FITC-avidin tracer (Figures 10B,C). Medium conditioned by cells sheared at the centre of the well (multidirectional flow) or by static cells had no effect.

Effects of heat treatment and size filtration, an unbiased proteomic analysis and ELISA of the medium identified follistatin-like 1 protein (FSTL1) as a likely mediator (Ghim et al., 2021b). Addition of recombinant FSTL1 reduced transport of quantum dot-labelled avidin across target endothelium in a concentration dependent manner (Figure 10C). It also reduced transendothelial transport of LDL itself (Figure 10D). (The fact that LDL and the quantum dot tracer were affected in same way is additional evidence that LDL is transported by receptor-independent, fluid-phase transcytosis.) It had no effect on transport of the smaller FITC-avidin tracer. FSTL1 and medium conditioned by cells swirled at the edge of the well also had anti-inflammatory effects, probably accounting for the results of the study of Ghim et al. (2018).

A preliminary investigation of signalling events downstream of FSTL1 (Ghim et al., 2021b) focused on the role of Bone Morphogenetic Protein 4 (BMP4) because this molecule is known to be expressed more highly in atheroprone than protected regions of the arterial tree, to be expressed more highly in the centre than the edge of swirling wells, and to be inhibited by FSTL1 (Sorescu et al., 2003; Geng et al., 2011; Souilhol et al., 2020). Exogenous recombinant human BMP4 increased transcytosis (Ghim et al., 2021b). FSTL1 blocked this barrier-disrupting effect, as did the BMP4 inhibitor noggin. However, FSTL1 was more effective: it reduced transport of the LDL-sized tracer below baseline levels, whereas noggin did not. Thus there appears to be at least one other pathway, in addition to blocking effects of BMP4, by which FSTL1 reduces permeability.

### Conclusion

The swirling well method in combination with substrate-binding tracers of different size and segmentation of cell growth has demonstrated that transWSS increases both paracellular and vesicular transport across endothelium, but by different mechanisms. The effect on LDL-sized particles may be caused by a lowered secretion of FSTL1 from the endothelium, which in turn causes a disinhibition of transcytosis.

## Limitations

The data and arguments presented in this review suffer from a number of limitations:

(i) There are no relevant transport data in people

It is difficult to measure transport of circulating macromolecules into the human arterial wall. Even when it has been accomplished (Tanimura et al., 1980), the fact that lesions are inevitably present makes the data of doubtful value: any spatial variation could be a result rather than a cause of disease, contrary to the situation in rabbits, where spontaneous disease is uncommon and vanishingly rare at the ages of greatest interest.

(ii) The non-human *in vivo* data derive from one site in one experimental model

The non-human *in vivo* data discussed above were obtained at side branches of the aorta in rabbits. White Carneau pigeons, which were once a popular model of atherosclerosis, and genetically modified mice, which are currently the most widely used, do not show the same age-related changes in lesion pattern at these anatomical locations (Richards and Weinberg, 2000; McGillicuddy et al., 2001).

Most conduit artery branches are bifurcations, where the parent vessel splits into two approximately equal daughter vessels, rather than T-branches like those seen in the aorta. TransWSS has been computed at the human carotid bifurcation (Gallo et al., 2016). There was no explicit comparison with disease, but high transWSS was seen on the flow divider and low transWSS was seen in the carotid sinus of many subjects, whereas disease prevalence shows broadly the opposite pattern (Grøttum et al., 1983). A formal evaluation of the spatial correlation is required.

Flow and LDL transport have been measured at the aorto-iliac bifurcation of mature rabbits by Berceli et al. (1990). LDL uptake was elevated in the lateral walls of the proximal segment of the daughter branches; high values of the OSI occurred in these segments when the vessels were perfused *in vitro*. McMillan (1985a,b) has speculated that highly multidirectional flow occurs at such locations, but the study of Berceli et al. assessed flow by Doppler techniques that could only measure velocity along the beam axis.

The relation of transWSS and disease has been assessed in coronary arteries, although not at branch points. A prospective study of human coronary arteries by Kok et al. (2019) found that transWSS was not significantly correlated with change in total plaque, fibrous tissue or fatty-fibrous tissue over time. TransWSS was significantly correlated with the increase in necrotic cores and dense calcification, but autocorrelation was not taken into account in the statistical analysis and significance will therefore have been overestimated. In a prospective study of porcine coronary arteries by Hoogendoorn et al. (2020), it is again clear that there was no relation between transverse WSS and disease progression (their Figure 1D). The association of lesion growth with other metrics such as the OSI and relative residence time may therefore reflect the fact that these metrics capture simple reversing flow as well as multidirectional flow. Neither study included cyclical motion of the coronary arteries when computing flow, and this may have introduced significant errors.

The pattern of albumin uptake around the left coronary bifurcation in rabbits showed significant changes with age but not the major reversal seen at aortic side branches (Staughton et al., 2007).

(iii) There is evidence for more than one mechanism and the evidence is self-contradictory

This limitation has been discussed at various points throughout the review. Briefly, some evidence suggests that there is a change with age in the mechanisms linking flow to transport, whilst other evidence suggests that the flow patterns themselves change with age. The former studies have largely supported the view that there is a change in the role of flow and nitric oxide, but there are inconsistencies; in particular, the effect of inhibiting NO production was unexpectedly small in mature rabbits *in vivo* (Staughton and Weinberg, 2004b), and the disappearance of the downstream pattern of transport in one study of anaesthetised rabbits (Staughton et al., 2001) is unexplained.

One set of studies suggesting a change with age in the pattern of flow is based on measuring nuclear elongation (Al-Musawi et al., 2004; Bond et al., 2011) while another is based on computational simulations of transWSS, a metric of multidirectionality (Peiffer et al., 2013a; Mohamied et al., 2015). However, elongation is not thought to depend on transWSS. The putative role of transWSS is questioned by the finding of apparently normal patterns of permeability in aortas perfused with a steady flow of buffer (Forster and Weinberg, 1997), where transWSS will everywhere be zero.

The discrepancies may be explained by technicalities: the restriction of early studies to measuring transport in a thin axial slice along the branch centreline; the use of tracers based on albumin, which likely enters the wall by both paracellular and transcellular routes but in different proportions in different preparations; and the incomplete understanding of the cause of endothelial nuclear shape.

(iv) Proteins other than FSTL1 may mediate effects of transWSS on transcytosis of LDL

An internal inconsistency in the *in vitro* study (Ghim et al., 2021b) identifying mediators is that although exogenous recombinant FSTL1 and medium conditioned by cells at the edge of the well both reduced transport of the LDL-sized tracer, much higher concentrations of exogenous FSTL1 were required than could be detected in the conditioned medium. This may reflect aberrant folding of the recombinant FSTL1 or differences in the experimental protocol (for example, cells were exposed to conditioned medium for longer than they were exposed to exogenous FSTL1), but another possibility is that additional mediator(s) were missed in the proteomic analysis. The cells used to condition medium were isolated from porcine aortas and the porcine proteome is less well annotated than the human one.

## Perspectives and Future Work

1 TransWSS is the mechanical factor that currently best explains the age-related patterns of permeability and lesion prevalence around side-branch ostia in the aorta. Further work is required in the following areas:
  i) to understand whether the same is true in other vessels. In particular, numerical simulations of coronary arteries that take into account cyclic variation in geometry may give different results from published studies that used static geometries, given the likely relation between radius of curvature, vortical structures and flow direction.
  ii) to refine the age-related maps of transWSS. Boundary conditions used in current studies may be insufficiently precise, given the probable dependence of transWSS patterns on posture and age-related changes in geometry.
  iii) to ascertain whether other shear metrics might show comparable age-related patterns. A key question is whether altering the extent of reverse flow in the side branch might give patterns of time average WSS similar to those already obtained for transWSS.
2 The age-related patterns of aortic permeability and lesion prevalence also strongly resemble patterns of endothelial nuclear elongation around branch ostia. That is currently unexplained, since high transWSS is not expected *a priori* to cause endothelial elongation. Important points are:
  i) are these expectations correct? the influence of transWSS on endothelial morphology needs systematic investigation.
  ii) does the apparent relation with nuclear elongation but not with time average WSS suggest that the pattern of time average WSS (commonly thought to determine elongation) has been mischaracterised in the simulations? This is related to point (1.iii), above.
3 TransWSS appears to suppress the endothelial secretion of atheroprotective mediators. A candidate mediator—FSTL1—has anti-inflammatory effects and reduces transcytosis, including fluid-phase transcytosis; it reduces permeability to LDL but not to albumin-sized tracers. This raises a number of issues:
  i) why does the pattern of disease correlate *in vivo* with the pattern of albumin transport, if albumin and LDL are transported by different routes, altered by different mediators? One solution to this, again requiring investigation, is that transWSS increases permeability to both types of macromolecule but through different pathways. Based on *in vivo* studies, transWSS might be affecting transport of albumin through an NO-dependent mechanism.
  ii) through which signalling pathways and by what effects on vesicle movement does FSTL1 alter LDL transport? A role for BMP4 has been demonstrated but there is also evidence that other pathways are involved. Studies of vesicular mechanics may require the development of new methods of dynamic, super-resolution microscopy.
  iii) are mediators other than FSTL1 involved? At present, it is known that any such mediators are likely to be proteins and to have sizes between 3 and 100 kDa. Use of human rather than porcine endothelial cells would permit more detailed proteomic studies to resolve this point.

## Conclusion

The relation between mechanical forces, transport properties of the arterial wall, and the localised occurrence of arterial disease has been investigated for over 100 years, and there has been intensive investigation of the role of WSS for over 50 years. The studies summarised above are consistent with 1) excessive net uptake of circulating macromolecules leading to a high prevalence of lesions, 2) excessive net uptake resulting from an excessive rate of influx into the wall, and 3) the pattern of influx and disease changing with age, explaining apparent discrepancies between human disease, animal disease and permeability. The data do not appear to support the simple low WSS hypothesis. However, the WSS characteristic that is responsible and the mechanisms linking WSS to transport have not been fully resolved. The problem is not that we do not have an explanation but that we have too many.

There may not be a single trigger. It is conceivable that endothelial cells have a “comfort zone” for a number of WSS metrics (and perhaps for other mechanical stresses as well), with disease developing when one or more metrics falls outside this range—for example, if WSS is too high or too oscillatory. That would complicate the establishment of causes, although at some point the pathways should converge on a single or small number of key properties, such as the transcytosis of LDL across endothelial cells.

An alternative to the comfort zone hypothesis is that the important WSS metric may be a complex or unexpected one, not revealed by early studies. Evidence has been presented that at least for aortic side branches, highly multidirectional near-wall flow is a trigger. Preliminary studies have identified a mediator—there may be others—linking this mechanical property to transcytosis of LDL. An interesting feature of this mediator is that it appears able to influence inflammatory pathways as well as permeability.

The view that atherogenesis is triggered by excessive entry of lipoproteins into the wall has been labelled the “response to influx” hypothesis (Weinberg, 2004). There is evidence that atherosclerosis develops as a “response to retention” of lipoproteins in the wall (Williams and Tabas, 1995), but the lipoproteins first have to get into the wall. The spatial correlations presented above suggest that it is the entry of the lipoproteins that is the rate-limiting step, and that any retention is simply proportional to it.

Statins have been successful at limiting the development of arterial disease. They work chiefly by lowering plasma cholesterol concentrations and, therefore, the entry of cholesterol into the wall. The same effect could be achieved by reducing permeability. An encouraging phenomenon is that the downstream lesions present in children regress with increasing age; the response to influx hypothesis attributes this to a decrease in permeability at that location, in the absence of marked changes in plasma cholesterol levels through the transitional years. A corollary is that reducing permeability could on its own be able to reduce adult disease. The two approaches—reducing plasma cholesterol concentrations and reducing arterial wall permeability—should have a multiplicative effect. A particular advantage of identifying a pathway that mediates between atherogenic flow and elevated permeability, rather than one which affects permeability globally, is that it could be used to reduce transport only where it is inappropriately elevated, and not to alter it in regions where permeability is instead matched to physiological need and sufficiently low that lesions do not develop.
