# Supplementary material for: Haemodynamic Wall Shear Stress, Endothelial Permeability and Atherosclerosis—A Triad of Controversy
Source: Front Bioeng Biotechnol. 2022 Mar 7;10:836680. doi: 10.3389/fbioe.2022.836680 (PMC8948426; doi:10.3389/fbioe.2022.836680)
Supplement: Supplementary file 1 [file Presentation1.pdf]

Supplementary Material

Supplementary Figure S1

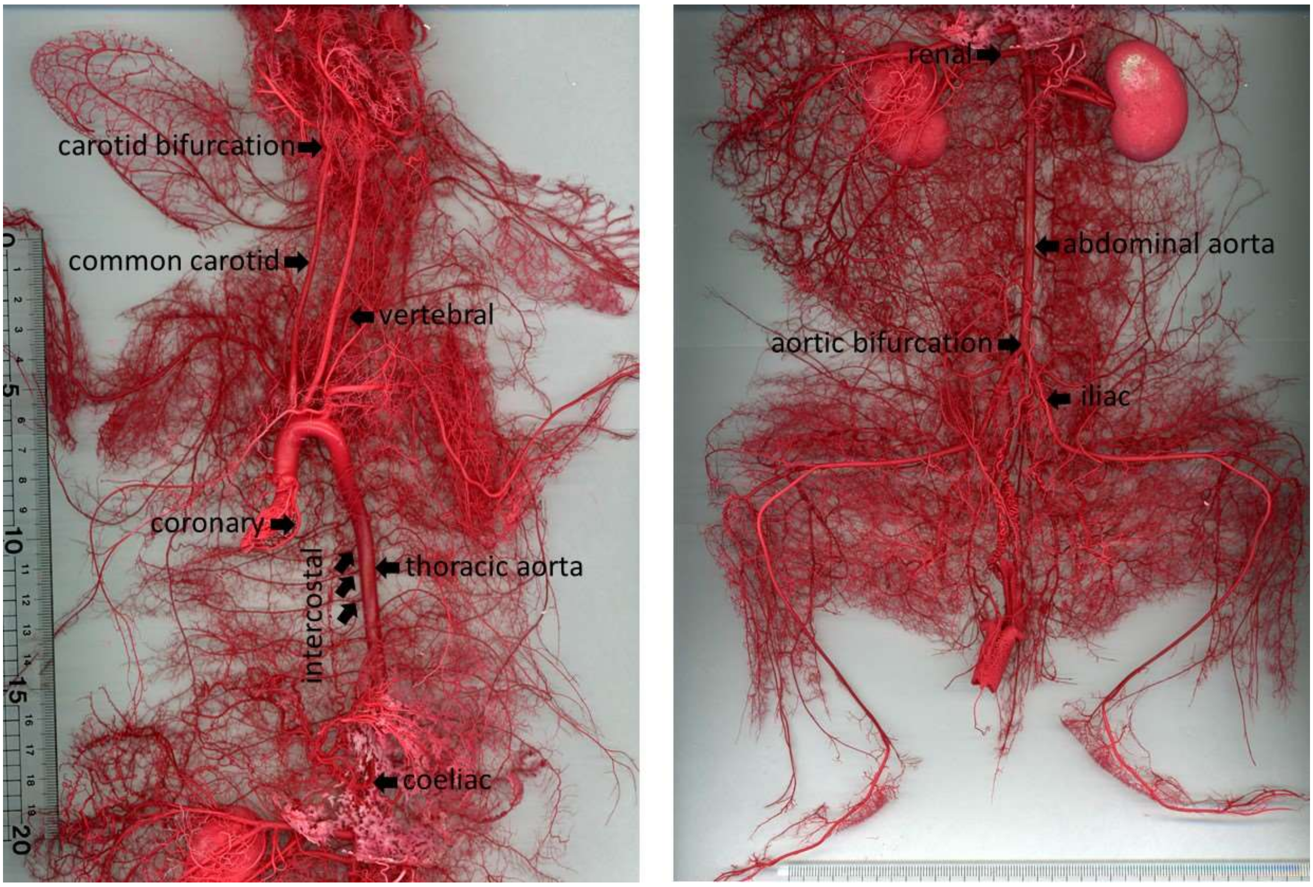

Figure S1. Ventral view of a corrosion cast of the rabbit arterial system (Left - cranial half; Right – caudal half) labelled to show arteries and bifurcations discussed in the paper. (Part of the renal vein has also been preserved.) Scale in cm.

Supplementary Figure S2

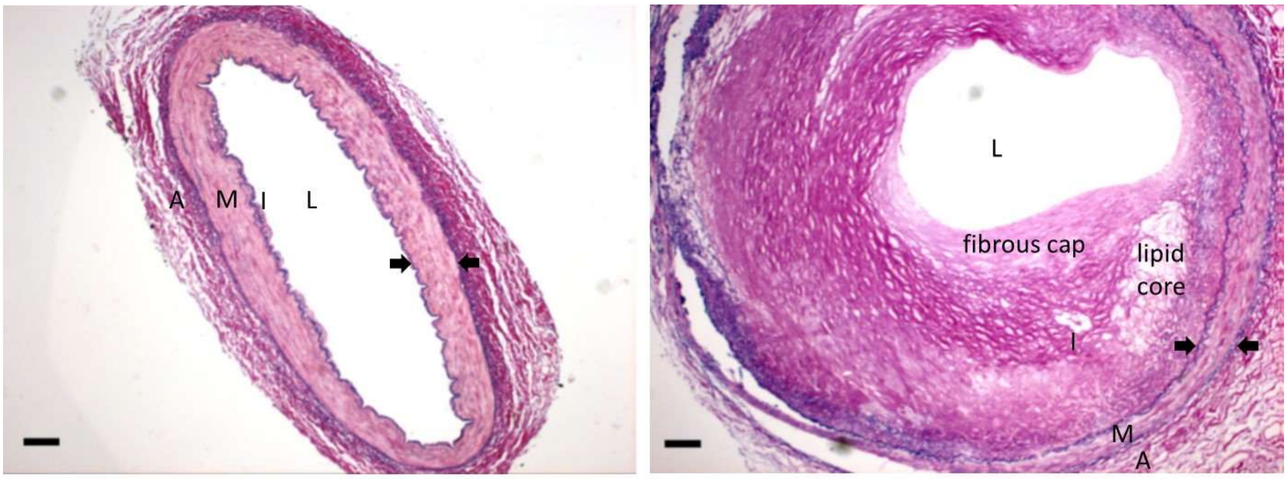

Figure S2. Transverse sections of (Left) a normal and (Right) a diseased coronary artery, treated with Elastic Van Gieson's stain to reveal elastin (blue/black) and collagen (red/pink) (Pang et al., 2021). The media (M) lies between the internal and external elastic lamellae (arrows), the adventitia (A) is external to the external elastic lamella, and the intima (I) internal to the internal elastic lamella. The lumen (L) is reduced in the diseased artery and the intima (which is little more than one cell thick in the normal vessel) is enlarged; the diseased intima contains a core of lipid and is surmounted by a fibrous cap. Bar = 100 µm

Pang, K. T., Ghim, M., Liu, C., Tay, H. M., Fhu, C. W., Chia, R. N., Qiu, B., Sarathchandra, P., Chester, A. H., Yacoub, M. H., Wilkinson, F. L., Weston, R., Warboys, C. M., Hou, H. W., Weinberg, P. D., & Wang, X. (2021). Leucine-Rich α-2-Glycoprotein 1 Suppresses Endothelial Cell Activation Through ADAM10-Mediated Shedding of TNF-α Receptor. *Frontiers in Cell and Developmental Biology*, 9, 706143. https://doi.org/10.3389/fcell.2021.706143
